# Identification and Phylogenetic Analysis of Heme Synthesis Genes in Trypanosomatids and Their Bacterial Endosymbionts

**DOI:** 10.1371/journal.pone.0023518

**Published:** 2011-08-10

**Authors:** João M. P. Alves, Logan Voegtly, Andrey V. Matveyev, Ana M. Lara, Flávia Maia da Silva, Myrna G. Serrano, Gregory A. Buck, Marta M. G. Teixeira, Erney P. Camargo

**Affiliations:** 1 Department of Microbiology and Immunology and the Center for the Study of Biological Complexity, Virginia Commonwealth University, Richmond, Virginia, United States of America; 2 Department of Parasitology, ICB, University of São Paulo, São Paulo, Brazil; Université Paris Sud, France

## Abstract

It has been known for decades that some insect-infecting trypanosomatids can survive in culture without heme supplementation while others cannot, and that this capability is associated with the presence of a betaproteobacterial endosymbiont in the flagellate's cytoplasm. However, the specific mechanisms involved in this process remained obscure. In this work, we sequence and phylogenetically analyze the heme pathway genes from the symbionts and from their hosts, as well as from a number of heme synthesis-deficient Kinetoplastida. Our results show that the enzymes responsible for synthesis of heme are encoded on the symbiont genomes and produced in close cooperation with the flagellate host. Our evidence suggests that this synergistic relationship is the end result of a history of extensive gene loss and multiple lateral gene transfer events in different branches of the phylogeny of the Trypanosomatidae.

## Introduction

Since the first attempts to cultivate trypanosomes of mammalian and other vertebrates, it became apparent that successful *in vitro* cultivation required red blood cells or blood derivatives in the culture media (for early literature see [1], [2]). Blood agar media were used to sustain cultures of trypanosomes for decades before heme-containing liquid media were developed [3]. The heme requirement also applied to the cultivation of trypanosomatids of insects for which hemoglobin or hemin had to be exogenously added to support growth [4].

In contrast, early observations [5] had shown that a peculiar trypanosomatid, *Strigomonas oncopelti*, did not require hemin or any heme-compound in its growth medium. Newton confirmed these findings by growing *S. oncopelti* in a very simple defined medium without heme-compounds [6], [7]. Thereafter, Newton and Horne [8] disclosed the presence of self-reproducing structures in the cytoplasm of *S. oncopelti*, which they called bipolar bodies. In fact, these bipolar bodies were the first endosymbionts described in trypanosomatids, and were later shown to be of betaproteobacterial nature [9]–[11].

Chang and collaborators [12]–[15] subsequently revealed the presence of endosymbionts in *Strigomonas* (* = Blastocrithidia*) *culicis* and demonstrated the ability of this flagellate to synthesize heme. The latter ability was absent in flagellates artificially cured of their symbionts by chloramphenicol treatment. It is interesting to note that, while the flagellate can live in culture without the endosymbiont, the opposite does not seem to be possible. Thus, the bacterial endosymbiont has not been successfully cultured outside of its host to date [16]. Other endosymbionts were later found in trypanosomatids for which autotrophy for heme was properly documented [4], [10], [17]–[19].

It became a commonly accepted inference that, since trypanosomatids required heme, they did not have the enzymatic equipment to make it *de novo* from amino acids. In contrast, since endosymbiont-carrying trypanosomatids did not require heme, their endosymbionts must have the genes and enzymes necessary to make heme. The inference, as sound as it was, remained to be proven.

**Figure 1:**
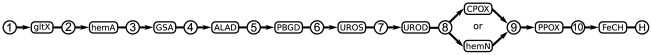
Synthetic pathway for heme. Enzymes: **gltX**, glutamyl-tRNA synthetase (EC:6.1.1.17); **hemA**, glutamyl-tRNA reductase (EC:1.2.1.70); **GSA**, glutamate-1-semialdehyde 2,1-aminomutase (EC:5.4.3.8); **ALAD**, aminolevulinic acid dehydratase (EC:4.2.1.24); **PBGD**, porphobilinogen deaminase (EC:2.5.1.61); **UROS**, uroporphyrinogen III synthase (EC:4.2.1.75); **UROD**, uroporphyrinogen III decarboxilase (EC:4.1.1.37); **CPOX**, coproporphyrinogen III oxidase (EC:1.3.3.3); **hemN**, oxygen-independent coproporphyrinogen III oxidase (EC:1.3.99.22); **PPOX**, protoporphyrinogen oxidase (EC:1.3.3.4); **FeCH**, ferrochelatase (EC:4.99.1.1). Compounds: **1**, L-glutamate; **2**, L-glutamyl-tRNA; **3**, glutamate-1-semialdehyde; **4**, aminolevulinic acid; **5**, porphobilinogen; **6**, hydroxymethylbilane; **7**, uroporphyrinogen III; **8**, coproporphyrinogen III; **9**, protoporphyrinogen IX; **10**, protoporphyrin IX; **H**, heme.

The synthesis of porphyrins, leading to chlorophyll in plants, or to heme in animals, starts with the production of aminolevulinic acid. This compound is produced by either one of two distinct pathways. In the C5 or Beale pathway, glutamic acid is the starting amino acid [20]. Photosynthetic organisms, most bacteria including Proteobacteria, and the Archaea use this heme biosynthetic pathway. In the Shemin pathway, glycine is the starting amino acid [21]. This pathway is present in animals, protozoa, fungi and Alphaproteobacteria. From aminolevulinic acid to heme, the enzymes are similar for all organisms (Fig. 1, depicted using the Beale pathway).

Recently, a review of heme functions and synthesis in eukaryotes was published, summing up the information on nutritional and enzymatic data of trypanosomatids and their symbionts ([22] and references therein). Their observations generally supported the concept that inability to synthesize heme is the default in trypanosomatids, except in those harboring endosymbionts. Thus, it was not surprising that the genomes of *T. cruzi* and *T. brucei* [23], [24] failed to show any of the genes of the heme pathway [22]. However, the genomes of some trypanosomatids e.g., *Leishmania* and *Crithidia*, contain the genes for the last three enzymes of the pathway (CPOX, PPOX and FeCH). Fittingly, these organisms grow when supplied with the immediate precursors of heme [25], [26]. Korený et al. [22] comparatively analyzed the genealogy of these genes and found them to group with the corresponding genes of the Gammaproteobacteria.

Presently, six trypanosomatid species are known to harbor symbionts [10]. They are referred to as SHTs (Symbiont Harboring Trypanosomatids) in contrast to regular trypanosomatids (RTs) lacking symbionts. To obtain direct evidence for the presence or absence and the genealogy of the heme-pathway genes, we sequenced to draft level the entire genomes of five SHTs: *Strigomonas oncopelti* (* = Crithidia oncopelti*), *S. culicis* (* = Blastocrithidia culicis*), *S. galati*, *Angomonas desouzai* ( = *C. desouzai*), and *A. deanei* ( = *C. deanei*), and fully sequenced the genomes of four of their betaproteobacterial symbionts, respectively: *Candidatus* Kinetoplastibacterium oncopeltii, *C*. K. blastocrithidii, *C*. K. galatii and *C.* K. crithidii, which are referred to as TPEs, Trypanosomatid Proteobacterial Endosymbionts [10]. The genomes of *C*. K. desouzaii, the TPE from *A. desouzai*, is currently being sequenced. For comparative analysis of heme-pathway genes, we also sequenced, to a draft level, the entire genomes of the RTs *Endotrypanum schaudinni, Leptomonas costaricensis, Crithidia acanthocephali*, *Phytomonas* sp., and *Herpetomonas muscarum*, and the bodonid *Parabodo caudatus* (* = Bodo caudatus*). The genomic sequences of these kinetoplastid and endosymbiont genomes will be reported elsewhere (in preparation). We also compared these genes with the available ones from *C. fasciculata* and *Leishmania* spp. In addition, gene sequences were phylogenetically compared with selected gene sequences of Alpha-, Beta-, and Gammaproteobacteria, plus other Bacteria and Eukaryota when necessary for phylogenetic resolution.

## Results

### Trypanosomatid and symbiont genes

Table 1 summarizes the results of the search for genes of the heme synthesis pathway in the genomes of symbiont bearing trypanosomatids (SHTs), regular trypanosomatids (RTs), and trypanosomatid proteobacterial endosymbionts (TPEs). As expected, all genomes, nuclear and endosymbiont, presented the gene for glutamyl-tRNA synthetase (gltX), which is essential for protein synthesis. In contrast, the genomes of all SHTs and RTs exhibited no evidence of the genes of the Shemin or Beale pathways for aminolevulinic acid synthesis. With the exception of CPOX, PPOX and FeCH, genes for the remaining enzymes for heme synthesis were also absent in these nuclear genomes. The gene encoding FeCH, the final enzyme in the heme biosynthesis pathway, was present in all flagellate genomes, whereas the CPOX and PPOX genes were present in *Crithidia, Leptomonas* and *Endotrypanum*, but absent in *Parabodo*, *Herpetomonas* and *Phytomonas*.

**Table 1:** Heme biosynthesis enzyme presence in the endosymbiotic bacteria and the Kinetoplastida analyzed in this work.

	gltX	hemA	GSA	ALAD	PBGD	UROS	UROD	CPOX^1^	hemN^1^	PPOX^2^	FeCH
*C*. K. blastocrithidii	+	+	+	+	+	+	+	-	+	-	+
*C*. K. crithidii	+	+	+	+	+	+	+	+	+	+	+
*C.* K. galatii	+	+	+	+	+	+	+	-	+	-	+
*C.* K. oncopeltii	+	+	+	+	+	+	+	-	+	-	+
*Angomonas deanei* 3	+	-	-	-	-	-	-	+	-	+	+
*Strigomonas culicis* 3	+	-	-	-	-	-	-	+	-	+	+
*S. galati* 3	+	-	-	-	-	-	-	+	-	+	+
*S. oncopelti* 3	+	-	-	-	-	-	-	+	-	+	+
*Crithidia acanthocephali* 4	+	-	-	-	-	-	-	+	-	+	+
*C. fasciculata* 4	+	-	-	-	-	-	-	+	-	+	+
*Endotrypanum schaudinni* 4	+	-	-	-	-	-	-	+	-	+	+
*Leptomonas costaricensis* 4	+	-	-	-	-	-	-	+	-	+	+
*Herpetomonas muscarum* 4	+	-	-	-	-	-	-	-	-	-	+
*Phytomonas sp.* 4	+	-	-	-	-	-	-	-	-	-	+
*Parabodo caudatus* 5	+	-	-	-	-	-	-	-	-	-	+

Enzyme name abbreviations are as in Figure 1. 1: enzymes capable of performing the same enzymatic reaction step in the pathway. 2: the PPOX putatively identified in *C.* K. crithidii is of a different family from the ones identified in the Kinetoplastida (see text); 3: symbiont-harboring trypanosomatids; 4: regular trypanosomatids; 5: bodonid.

**Figure 2:**
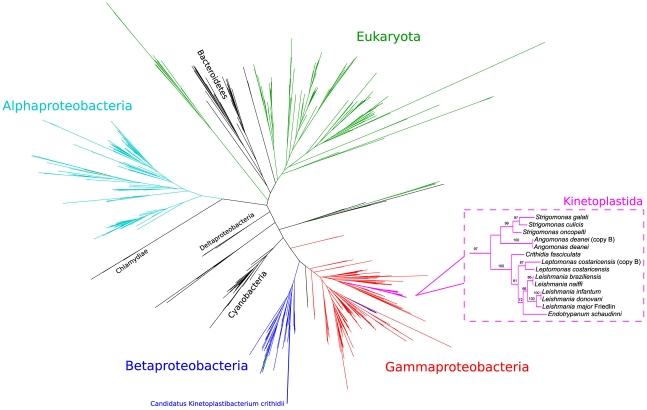
Maximum-likelihood phylogeny of coproporphyrinogen III oxidase. Inset shows details of the tree in the Kinetoplastida clade. Numbers on the branches represent bootstrap support from analysis of 100 pseudo-replicates (only values of 50 or more shown). Colors represent some of the major taxa.

In contrast to the nuclear genomes of these flagellates, the genomes of their endosymbionts (TPEs) bear the genes of the Beale pathway for the synthesis of aminolevulinic acid. With the exception of the CPOX and PPOX genes, all of the genes for heme synthesis from levulinic acid to heme were also present in all TPEs. Instead of CPOX, the symbiont genomes carry the gene for an oxygen-independent coproporphyrinogen oxidase (hemN) that can, in principle, catalyze the synthesis of protoporphyrinogen. The symbionts of *A. deanei* (*C*. K. crithidii) and *A. desouzai* (*C*. K. desouzaii, not shown) possess a CPOX gene in addition to the hemN gene (Table 1, Figs. 2 and S1). The *Angomonas* TPEs also present a gene that is similar to a recently characterized novel PPOX called hemJ [27], while the other TPEs do not present this gene.

We have also screened the genomes of *Crithidia fasciculata* and *Leishmania* spp. available in public databases. We found that genes encoding PPOX, CPOX and FeCH are present in the genomes of *Leishmania major*, *L. braziliensis*, *L. infantum*, *L. mexicana*, *L. tarentolae*, and *C. fasciculata*. Since these species are considered to be closely related [28], it was not surprising that the genes encoding these three genes were quite similar in sequence (data not shown). Therefore, for efficiency, we did not include all of these genes in the analyses below.

### Phylogeny of the genes for heme synthesis

As described above, the gene for glutamyl-tRNA synthase, which is essential for tRNA metabolism, was found in the nuclear genomes of all the trypanosomatids and symbionts examined. Our preliminary phylogenies involving a wide selection of organisms showed that the gene belongs to a eukaryotic lineage in all trypanosomatid nuclear genomes (data not shown) and its phylogeny was therefore not explored further in this work. In contrast, in the symbionts, the glutamyl-tRNA synthase gene, like those of the majority of the symbiont genes (in preparation), is of betaproteobacterial origin (Fig. S2).

The gene for FeCH is also present in the genomes of SHTs, TPEs and all RTs, but has different phylogenies: in the genomes of the TPEs, the gene is of betaproteobacterial descent, whereas in the genomes of the SHTs and RTs the FeCH gene is apparently descended from three different lineages of Gammaproteobacteria (Figs. S3,S4,S5). Therefore, SHTs have two sets of genes for FeCH, one of apparent gammaproteobacterial origin in the nucleus, and another of betaproteobacterial descent in the symbionts.

Although the FeCH genes from both the RTs and SHTs group with the Proteobacteria, their exact position is unclear. In general, the Gammaproteobacteria were split in several subgroups around the tree, with insignificant bootstrap support values uniting the different groups. The FeCH gammaproteobacterial genes from *Parabodo* and *Phytomonas* seem to have different phylogenetic histories (Fig. S5): the *Parabodo* gene groups with *Moritella* whereas the *Phytomonas* gene groups with *Providencia* and other Gammaproteobacteria, although with low support in both cases (48 and 43 respectively).

As described above, genes for CPOX and PPOX are present in *Angomonas*, *Strigomonas*, and the *Leishmania* group (*Leishmania*, *Leptomonas*, *Crithidia*, and *Endotrypanum*). Phylogenies for both genes clearly show them grouped within the Gammaproteobacteria (Figs. 2, S6, and S7). While the Kinetoplastida form a monophyletic group with a high bootstrap value for CPOX (Fig. S6), they are divided in two well supported groups for PPOX: one group involving the *Leishmania* group, and the other involving *Angomonas* and *Strigomonas* (Fig. S7).

**Figure 3:**
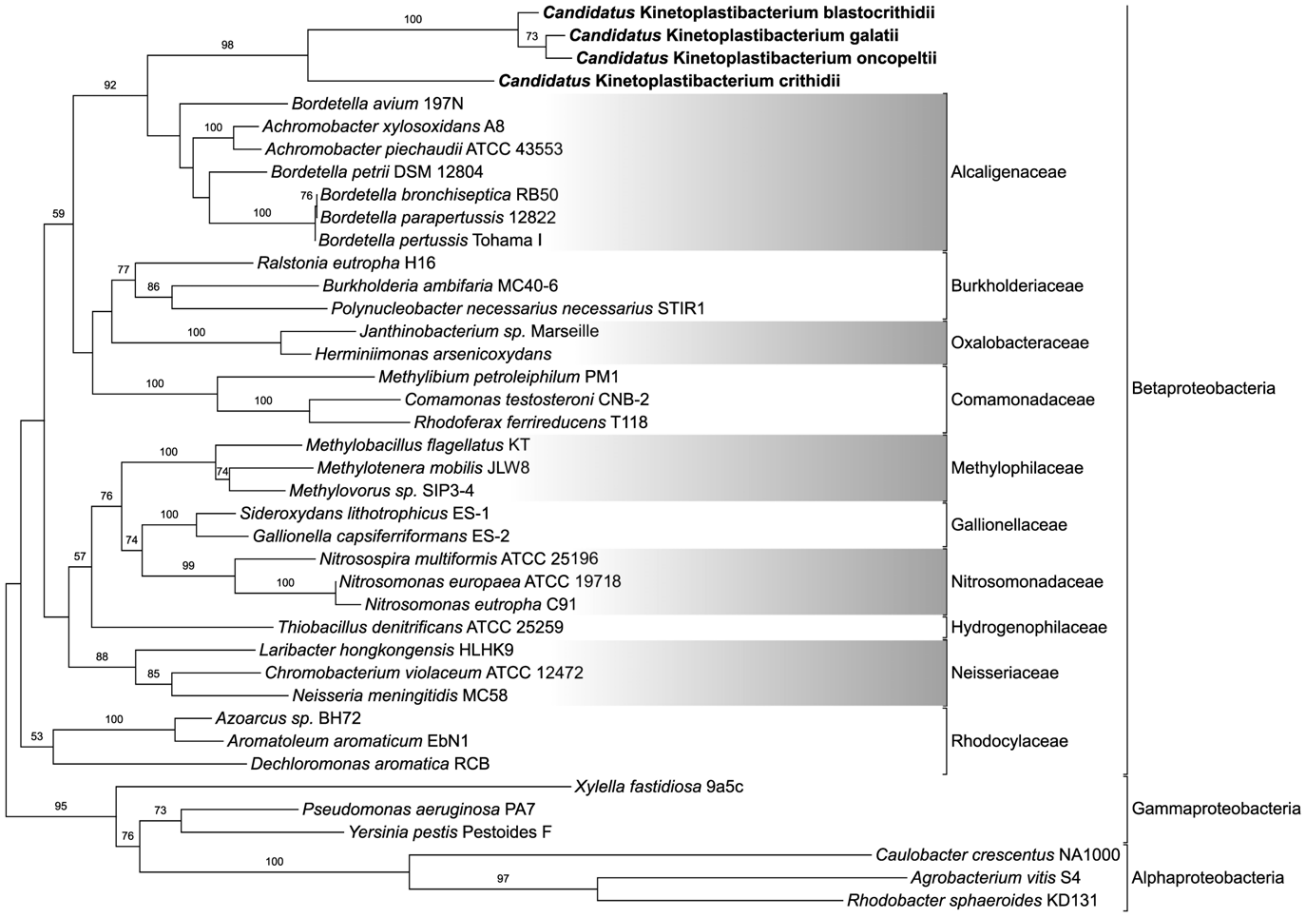
Maximum-likelihood phylogeny of porphobilinogen deaminase from representative Betaproteobacteria, the endosymbiotic *C.* Kinetoplastibacterium sp., and Alpha- and Gammaproteobacteria as outgroups. Numbers on the branches represent bootstrap support from analysis of 100 pseudo-replicates (only values of 50 or more shown).

The genes of the heme pathway of TPE genomes (*C*. K. crithidii, *C.* K. oncopeltii, *C.* K. galatii and *C.* K. blastocrithidii) were compared with the corresponding genes of selected Alpha-, Beta- and Gammaproteobacteria. An example of the phylogenetic pattern of all genes present in the symbionts is in Fig. 3, which shows the inferred phylogenetic tree for the PBGD gene. Similar to the PBGD gene of the symbionts, phylogenies of hemA, GSA, ALAD, UROS, UROD and hemN genes (abbreviations as in Fig. 1) always show the TPE genes as a sister group of the corresponding genes of the Alcaligenaceae family, e.g. *Bordetella* spp. and *Achromobacter* spp. The genes of the TPEs and the Betaproteobacteria constitute a monophyletic branch supported by bootstraps varying from 85% to 100%, more often closer to the latter (Figs. 3, S2, S4, and S8,S9,S10,S11,S12,S13). The heme pathway genes of the TPEs are clearly distant from the equivalent genes of Alpha- and Gammaproteobacteria. The hemJ-like PPOX genes in *C.* K. crithidii and *C.* K. desouzaii are quite similar to their homologs in other Betaproteobacteria, in particular in *Bordetella* and *Achromobacter* (data not shown).

## Discussion

As previously reported, the symbiotic association between ancestral trypanosomatids and Betaproteobacteria was probably the result of a single event that occurred in the late Cretaceous Period [9], [10]. In the tens of millions of years of co-evolution since then, both symbiont and host have adapted to each other. As in other endosymbiotic associations [29], [30], the genomes of the TPEs were reduced in size and underwent a dramatic lowering of their GC compositions to about 30% (in preparation). In contrast, the genomes of *Bordetella* and *Achromobacter*, which are closely related to the endosymbionts described herein, exhibit 61–68% GC.

In this paper, all of the genes required for heme synthesis were found, in the combination between the host and symbiont genomes, and phylogenetically analyzed. Our data show that the genomes of SHTs and some RTs seem to have incorporated at least three gammaproteobacterial genes that encode enzymes required for heme biosynthesis. Thus, a horizontal transfer of these genes from a gammaproteobacterial organism to the nuclear genome of the progenitor protozoan probably occurred prior to the event resulting in the stabilization of the betaproteobacterial endosymbiont. In contrast, the genomes of the TPEs have a nearly complete set of genes of betaproteobacterial descent for heme synthesis, suggesting that these genes were present in a betaproteobacterial progenitor to the endosymbionts.

As previously inferred by nutritional and enzymatic data ([22] and references therein), a number of trypanosomatids exhibit a biochemical arsenal that is complemented by their betaproteobacterial endosymbionts. For at least one of these biochemical pathways, heme biosynthesis, we have defined herein the genetic basis of this interaction; i.e. the endosymbiont supplies enzymatic functions for heme biosynthesis that are absent from the nuclear genome of the host, and possibly vice-versa in the case of PPOX. We have also found that the heme synthetic genes have been the stage of an intricate history of multiple symbiotic and lateral gene transfer (LGT) events.

Data on the genomic structure of the TPEs (in preparation) and phylogenetic analyses of heme genes described herein strongly agree with previous findings placing the TPEs in the Betaproteobacteria division, closest to *Bordetella* in the family Alcaligenaceae [9], [10]. However, our present data do not permit further advancement of the taxonomy of TPEs, for which multiple gene analyses are in progress.

Regardless of the incompleteness of taxonomic information, it is clear from our observations that the endosymbiont genomes are carrying the bulk of the genes for heme synthesis in the SHTs analyzed. However, some differences between the symbionts of *Strigomonas* (*C*. K. blastocrithidii, *C*. K. galatii and *C*. K oncopeltii) and the symbionts of *Angomonas* (*C*. K. crithidii and *C*. K. desouzaii) were found. Thus, the genomes of all symbionts have a hemN gene for processing coproporphyrinogen, but the symbionts of *Angomonas* have a copy of a CPOX gene that can perform a similar function. At present, it is not known whether only one or both of these genes are functional. Both are of betaproteobacterial origin (Figs. S1 and S13), and absence of CPOX in the *Strigomonas* symbionts may have resulted from its loss in a progenitor. However, we cannot exclude *a priori* the possibility that an additional betaproteobacterial LGT is responsible for the presence of the CPOX gene in *Angomonas* symbionts. It is important to remember that the trypanosomatid nuclear genomes also contain their own copy of CPOX, which might also be active, as discussed below.

As previously reported [22], gammaproteobacterial CPOX, PPOX and FeCH are present in *Leishmania* and *Crithidia*. Our phylogenetic analyses of the newly sequenced trypanosomatids (*Leptomonas*, *Endotrypanum*, *Angomonas*, and *Strigomonas*) and the previously reported gene sequences entirely support these observations (Figs. 2, S3, S5,S6,S7). In contrast, we were unable to identify genes for CPOX or PPOX in *Herpetomonas*, *Phytomonas*, or *Parabodo* (Table 1). We also showed that a PPOX gene is present in all the SHTs, and possibly two of the four TPE genomes. While the flagellate genes are of clear gammaproteobacterial descent, the genes for PPOX are present in two distinct clades (Fig. S7), one containing the SHTs and the other containing the RTs, and may result from distinct LGT events. The situation is more complex in the TPEs. The identification of PPOX in organisms across the tree of life is a surprisingly contentious issue compared to the other, well conserved enzymes of the pathway; the identification of the gene responsible for the conversion of protoporphyrinogen to protoporphyrin is still elusive in many groups [31]. There are currently at least three identified enzyme types presenting evidence of PPOX activity, and at least a fourth type is a possibility [27]. We have identified a hemJ-like protein in *C*. K. crithidii and *C*. K. desouzaii. We have not performed phylogenetic analyses for these hemJ-like proteins, which are very similar in sequence to the putative homologs from bacteria of the Alcaligenaceae family, including *Bordetella* and *Achromobacter*. Previously published evidence [27] shows that hemJ from *Bordetella* and other Betaproteobacteria group together in their phylogenetic tree. Further studies will be necessary to confidently point to the gene, if any, performing the PPOX function in the TPEs. It is also possible that this function is being performed, as discussed in the case of CPOX, by the PPOX encoded by the SHT's genome. Freed from the selective pressure to keep PPOX, the TPEs seem to be in the midst of a reductive genomic evolution event where one lineage (the TPEs from *Strigomonas*) has already lost its copy, while another (the TPEs from *Angomonas*) still has not.

Another intriguing observation concerns the origins of FeCH genes. In SHTs, the FeCH located in the TPE genomes is of clear betaproteobacterial descent (Fig. S4). The FeCH genes of the flagellates however group together with high bootstrap support, but their exact placement among the Gammaproteobacteria is not clear due to low bootstrap support at deeper levels of the phylogeny (Fig. S5). The FeCH genes of *Parabodo* and *Phytomonas*, on the other hand, are in different parts of the gammaproteobacterial tree and might result from distinct LGT events. While the *Parabodo* gene gets a reasonable branch length linking it to *Moritella* in phylogenetic trees, the FeCH from *Phytomonas* is linked to *Providencia* by a much longer branch, which can be a problematic situation in phylogenetic reconstruction due to the long branch attraction effect [32]. We believe that better taxonomic sampling of gammaproteobacterial sequences more closely related to the FeCH of *Phytomonas* would be a more confident way to define its phylogenetic position by breaking these long branches. It might also be possible that this gene presents too much variation to permit the identification of older phylogenetic signals, in which case its exact phylogenetic patterns may never be confidently ascertained for older divergences.

In conclusion, the genomic data presented herein strongly support previous biochemical observations concerning the heme requirements of trypanosomatids and the heme autotrophy exhibited by trypanosomatids harboring endosymbionts. In the partnership between the trypanosomatid hosts and their symbionts, the genes for the synthesis of heme are provided mostly by the symbiont genomes, requiring a close cooperation between the flagellates and their endosymbionts. Such cooperation and co-evolution are the final results of an extensive history of endosymbiosis, gene loss, and multiple lateral gene transfer events. Further studies on the earlier branching of trypanosomatids are necessary to pinpoint the place in its phylogeny where the ancestral eukaryotic heme synthesis genes were first lost and where they were replaced in some lineages by proteobacterial genes through LGT events.

## Materials and Methods

### Organisms and growth conditions

The genomes of the following organisms were sequenced: *Parabodo caudatus* ATCC 30905, *Angomonas deanei* (TCC036E), *Angomonas desouzai* (TCC079E), *Strigomonas culicis* (TCC012E), *Strigomonas galati* (TCC219), *Strigomonas oncopelti* (TCC290E), *Herpetomonas muscarum* (TCC001E), *Crithidia acanthocephali* (TCC037E), *Endotrypanum schaudinni* (TCC224), *Leptomonas costaricensis* (TCC169E), and *Phytomonas* sp. from *Jatropha macrantha* (TCC066E). These organisms are cryopreserved at the Trypanosomatid Culture Collection of the University of São Paulo, TCC-USP, with the exception of *Parabodo caudatus*, available from the American Type Culture Collection.

Except where noted below, flagellates were grown in LIT media [3] supplemented with 2% FBS (Fetal Bovine Serum). *Endotrypanum* was grown in TC100 medium (Cultilab, Brazil), which is similar to Grace's Medium (Gibco), supplemented with 2% FBS at 28°C. *Parabodo* was grown on rye grass cerophyll infusion (Sonneborn's Paramecium medium, ATCC #802) at 25°C. Live *Enterobacter aerogenes* (ATCC 13048) was added as food for the flagellate. To minimize the amount of bacterial contamination in the samples to be examined, cultures were washed several times in PBS and finally fractionated twice in a Percoll/0.25M sucrose discontinuous gradient (80% to 10% percoll/sucrose, layered in 10% dilution steps), spun 30 minutes at 3200 x g. The bacteria-depleted, 60–80% gradient fraction was collected, further washed and used as the source of flagellates.

### DNA extraction and kDNA depletion

Genomic DNA was extracted from cultured trypanosomatids using the phenol/chloroform method [33]. To minimize the amount of kinetoplast DNA present in the sequencing, total genomic DNA (∼50 µg in 300 µL) from each trypanosomatid isolate was loaded onto a 0.8% low melting agarose gel in 0.5 x TAE buffer. Electrophoresis was performed at low voltage, not exceeding 4 V/cm, for 4–5 hours. After electrophoresis, the gel was washed in distilled water for ∼1 hour for destaining and buffer removal. The genomic band was carefully cut out and purified by treatment with beta-agarase I (NEB, cat. # M0392S), as described by the manufacturer. The DNA was precipitated with ethanol in 0.3 M sodium acetate, washed with 70% ethanol, dried, and finally dissolved in 100–120 µL TE, pH 8.0. Our results (not shown) indicate that this genomic DNA enrichment protocol reduces the proportion of kDNA in the sample to less than 5%, and therefore provides a template that yields coverage of both genomic and kDNA.

### DNA sequencing, assembly, and gene calling

After kDNA depletion, 5 µg of total genomic DNA were sequenced to an estimated coverage of between 13 and 19x (for an estimated genome size of 35 million bases) using standard pyrosequencing shotgun methodology according to Roche 454 protocols. Resulting reads were assembled by Roche's Newbler software (version 2.3). For SHTs, contigs were segregated into endosymbiont- or host-derived sets by similarity of their sequences to currently available betaproteobacterial genomes; after closing of the endosymbiont genomes, any contigs not matching the finished TPEs were returned to the host set. Closing of the bacterial chromosome sequences was performed by multiplex long-range PCR using primers based on the ends of the longest contigs, followed by capillary sequencing of the resulting products and manual gap closing. Previously determined *Crithidia fasciculata* genomic draft-level sequences were kindly provided by Dr. Stephen M. Beverley and produced by The Genome Institute at Washington University School of Medicine in St. Louis and can be obtained from http://tritrypdb.org/ [34].

Genes from the newly sequenced SHTs plus RTs were identified by similarity to their *Leishmania* homologs, and translation start and stop sites were verified manually by comparative analysis with other known sequences and protein domains. Genes from TPEs were first called using Glimmer [35], and then manually checked for completeness and adequate translation start and stop sites, as above. GenBank accession numbers for all sequences generated in this work are available in the supporting material (Tables S1,S2,S3,S4,S5,S6,S7,S8,S9,S10,S11).

### Ortholog selection and phylogenetic analyses

All analyses were performed at the protein sequence level. Candidate orthologs for the genes of the heme pathway were detected by two different approaches: a) for genes to be analyzed in phylogenies with large taxon sampling across the tree of life, similarity searches of each gene against NCBI's non-redundant protein database were used, collecting candidate orthologs from several organisms; b) for genes that were to be analyzed solely in the Betaproteobacteria, we used KEGG [36] enzyme assignments for Betaproteobacteria representative of all currently sequenced families, restricting the number of organisms per family to a maximum of three by using a phylogeny of the group as a guide [37]. As an exception, the genomes of all available members of the Alcaligenaceae family were analyzed since there was previous evidence of this being the family to which the TPEs belong [9], [10]. Whenever KEGG assignments were not available, putative orthologs were identified by similarity searches performed against relevant genomes. All putative orthologs were also analyzed by RPSBLAST searches [38] against the Conserved Domains Database [39] to identify domains characteristic of their function, to minimize the possibility of selecting a gene based only on partial domain similarity or convergence due to partially similar functionality.

Selected sequences were aligned by ClustalW2 [40] and visually analyzed for very divergent sequences, which were then separately re-analyzed to confirm whether they were not spuriously classified as an ortholog (e.g. due to similarity in protein domains), as described above. Such distant sequences were removed from the analysis, and remaining sequences were realigned as above. Alignments were then manually edited for refinement. Taxonomic affiliation and accession numbers for all sequences included in the final phylogenetic analyses are available in the supporting material (Tables S1,S2,S3,S4,S5,S6,S7,S8,S9,S10,S11).

Maximum likelihood phylogenetic inferences were performed by RAxML v. 7.2.8 [41] on a Linux computer cluster using between 4 and 45 processor cores, depending on the number of taxa in the alignment. The substitution model used was the empirical WAG model [42], with gamma-distributed heterogeneity rate categories and estimated empirical residue frequencies (model PROTGAMMAWAGF). Two hundred different best tree searches were performed for each alignment, and the tree with best likelihood found was kept. RAxML's rapid bootstrap was performed with 100 pseudo-replicates and support is only shown in the phylogenies for branches with support of at least 50. With the exception of the radial trees, all other trees were drawn and basically formatted by TreeGraph2 [43]. Further cosmetic adjustments were done using the Inkscape vector image editor (http://inkscape.org/). Radial trees were drawn in Dendroscope [44].

## Supporting Information

Figure S1 **Maximum-likelihood phylogeny of coproporphyrinogen III oxidase in the Betaproteobacteria.** This is a detailed view derived from the radial tree presented in Figure 2. Numbers in black circles mark betaproteobacterial families. Other numbers on branches show bootstrap support from analysis of 100 pseudo-replicates (only 50 or greater shown).
(EPS)

Figure S2 **Maximum-likelihood phylogeny of glutamyl-tRNA synthetase from representative Betaproteobacteria, and Alpha- and Gammaproteobacteria as outgroups.** Numbers on branches represent bootstrap support from analysis of 100 pseudo-replicates (only 50 or greater shown).
(EPS)

Figure S3 **Maximum-likelihood phylogeny of ferrochelatase.** Colored branches mark some of the major clades present. For detailed depiction of the Beta- and Gammaproteobacteria, see Figures S3 and S4.
(EPS)

Figure S4 **Maximum-likelihood phylogeny of ferrochelatase in the Betaproteobacteria.** This is a detailed view derived from the radial tree presented in Figure S2. Numbers on branches show bootstrap support (only 50 or greater shown) from analysis of 100 pseudo-replicates .
(EPS)

Figure S5 **Maximum-likelihood phylogeny of ferrochelatase in the Gammaproteobacteria.** This is a detailed view derived from the radial tree presented in Figure S2. Numbers on branches show bootstrap support (only 50 or greater shown) from analysis of 100 pseudo-replicates .
(EPS)

Figure S6 **Maximum-likelihood phylogeny of coproporphyrinogen III oxidase in the Gammaproteobacteria and the Kinetoplastida.** This is a detailed view derived from the radial tree presented in Figure 2. The Kinetoplastida names are drawn in blue. Numbers on branches show bootstrap support from analysis of 100 pseudo-replicates (only 50 or greater shown).
(EPS)

Figure S7 **Maximum-likelihood phylogeny of protoporphyrinogen oxidase from representative Gammaproteobacteria, and the Kinetoplastida.** Numbers on branches represent bootstrap support from analysis of 100 pseudo-replicates (only 50 or greater shown).
(EPS)

Figure S8 **Maximum-likelihood phylogeny of glutamyl-tRNA reductase from representative Betaproteobacteria, and Alpha- and Gammaproteobacteria as outgroups.** Numbers on branches represent bootstrap support from analysis of 100 pseudo-replicates (only 50 or greater shown).
(EPS)

Figure S9 **Maximum-likelihood phylogeny of glutamate-1-semialdehyde 2,1-aminomutase from representative Betaproteobacteria, and Gammaproteobacteria as outgroups.** Numbers on branches represent bootstrap support from analysis of 100 pseudo-replicates (only 50 or greater shown).
(EPS)

Figure S10 **Maximum-likelihood phylogeny of aminolevulinic acid dehydratase from representative Betaproteobacteria, and Alpha- and Gammaproteobacteria as outgroups.** Numbers on branches represent bootstrap support from analysis of 100 pseudo-replicates (only 50 or greater shown).
(EPS)

Figure S11 **Maximum-likelihood phylogeny of uroporphyrinogen III synthase from representative Betaproteobacteria, and Alpha- and Gammaproteobacteria as outgroups.** Numbers on branches represent bootstrap support from analysis of 100 pseudo-replicates (only 50 or greater shown).
(EPS)

Figure S12 **Maximum-likelihood phylogeny of uroporphyrinogen III decarboxilase from representative Betaproteobacteria, and Alpha- and Gammaproteobacteria as outgroups.** Numbers on branches represent bootstrap support from analysis of 100 pseudo-replicates (only 50 or greater shown).
(EPS)

Figure S13 **Maximum-likelihood phylogeny of oxygen-independent coproporphyrinogen III oxidase from representative Betaproteobacteria, and Alpha- and Gammaproteobacteria as outgroups.** Numbers on branches represent bootstrap support from analysis of 100 pseudo-replicates (only 50 or greater shown).
(EPS)

Table S1 **Proteins utilized in the phylogenetic analysis of glutamyl-tRNA synthetase (gltX) and the respective organism names.**
(PDF)

Table S2 **Proteins utilized in the phylogenetic analysis of glutamyl-tRNA reductase (hemA) and the respective organism names.**
(PDF)

Table S3 **Proteins utilized in the phylogenetic analysis of glutamate-1-semialdehyde 2,1-aminomutase (GSA) and the respective organism names.**
(PDF)

Table S4 **Proteins utilized in the phylogenetic analysis of aminolevulinic acid synthase (ALAD) and the respective organism names.**
(PDF)

Table S5 **Proteins utilized in the phylogenetic analysis of porphobilinogen deaminase (PBGD) and the respective organism names.**
(PDF)

Table S6 **Proteins utilized in the phylogenetic analysis of uroporphyrinogen III synthase (UROS) and the respective organism names.**
(PDF)

Table S7 **Proteins utilized in the phylogenetic analysis of uroporphyrinogen III decarboxilase (UROD) and the respective organism names.**
(PDF)

Table S8 **Proteins utilized in the phylogenetic analysis of oxygen-independent coproporphyrinogen III oxidase (hemN) and the respective organism names.**
(PDF)

Table S9 **Proteins utilized in the phylogenetic analysis of coproporphyrinogen III oxidase (CPOX) and the respective organism names.**
(PDF)

Table S10 **Proteins utilized in the phylogenetic analysis of protoporphyrinogen oxidase (PPOX) and the respective organism names.**
(PDF)

Table S11 **Proteins utilized in the phylogenetic analysis of ferrochelatase (FeCH) and the respective organism names.**
(PDF)
