# Supplementary material for: Identification and Phylogenetic Analysis of Heme Synthesis Genes in Trypanosomatids and Their Bacterial Endosymbionts
Source: PLoS One. 2011 Aug 10;6(8):e23518. doi: 10.1371/journal.pone.0023518 (PMC3154472; doi:10.1371/journal.pone.0023518)
Supplement: Table S1 — Proteins utilized in the phylogenetic analysis of glutamyl-tRNA synthetase (gltX) and the respective organism names. (PDF) [file pone.0023518.s014.pdf]

Accession number	Organism
**JF756629**	*Candidatus* Kinetoplastibacterium blastocrithidii
**JF756630**	*Candidatus* Kinetoplastibacterium crithidii
**JF756631**	*Candidatus* Kinetoplastibacterium galatii
**JF756632**	*Candidatus* Kinetoplastibacterium oncopeltii
ZP_06685724.1	*Achromobacter piechaudii* ATCC 43553
YP_003976719	*Achromobacter xylosoxidans* A8
YP_002550790	*Agrobacterium vitis* S4
YP_160940.1	*Aromatoleum aromaticum* EbN1
YP_932983.1	*Azoarcus sp.* BH72
YP_787472	*Bordetella avium* 197N
NP_890905.1	*Bordetella bronchiseptica* RB50
NP_886048.1	*Bordetella parapertussis* 12822
NP_881555.1	*Bordetella pertussis* Tohama I
YP_001629191.1	*Bordetella petrii* DSM 12804
YP_001808655.1	*Burkholderia ambifaria* MC40-6
YP_002517355	*Caulobacter crescentus* NA1000
NP_901607.1	*Chromobacterium violaceum* ATCC 12472
YP_003277450.1	*Comamonas testosteroni* CNB-2
YP_284541.1	*Dechloromonas aromatica* RCB
YP_003847394.1	*Gallionella capsiferriformans* ES-2
YP_001099291.1	*Herminiimonas arsenicoxydans*
YP_001352803.1	*Janthinobacterium sp.* Marseille
YP_002795843.1	*Laribacter hongkongensis* HLHK9
YP_001021340.1	*Methylibium petroleiphilum* PM1
YP_545699.1	*Methylobacillus flagellatus* KT
YP_003048325.1	*Methylotenera mobilis* JLW8
YP_003051465.1	*Methylovorus sp.* SIP3-4
NP_273069.1	*Neisseria meningitidis* MC58
NP_841659.1	*Nitrosomonas europaea* ATCC 19718
YP_746740.1	*Nitrosomonas eutropha* C91
YP_412297.1	*Nitrosospira multiformis* ATCC 25196
YP_001797707.1	*Polynucleobacter necessarius necessarius* STIR1
YP_001347374.1	*Pseudomonas aeruginosa* PA7
YP_726863.1	*Ralstonia eutropha* H16
YP_002524658	*Rhodobacter sphaeroides* KD131
YP_523928.1	*Rhodoferax ferrireducens* T118
YP_003524162.1	*Sideroxydans lithotrophicus* ES-1
YP_315492.1	*Thiobacillus denitrificans* ATCC 25259
NP_298112.1	*Xylella fastidiosa* 9a5c
YP_001163444.1	*Yersinia pestis* Pestoides F

GenBank accession numbers in bold typeface were sequenced in this work.
