# Supplementary material for: Identification and Phylogenetic Analysis of Heme Synthesis Genes in Trypanosomatids and Their Bacterial Endosymbionts
Source: PLoS One. 2011 Aug 10;6(8):e23518. doi: 10.1371/journal.pone.0023518 (PMC3154472; doi:10.1371/journal.pone.0023518)
Supplement: Table S2 — Proteins utilized in the phylogenetic analysis of glutamyl-tRNA reductase (hemA) and the respective organism names. (PDF) [file pone.0023518.s015.pdf]

Accession number	Organism
**JF756625**	*Candidatus* Kinetoplastibacterium blastocrithidii
**JF756626**	*Candidatus* Kinetoplastibacterium crithidii
**JF756627**	*Candidatus* Kinetoplastibacterium galatii
**JF756628**	*Candidatus* Kinetoplastibacterium oncopeltii
ZP_06684613.1	*Achromobacter piechaudii* ATCC 43553
YP_003981972	*Achromobacter xylosoxidans* A8
YP_157646.1	*Aromatoleum aromaticum* EbN1
YP_932484.1	*Azoarcus sp.* BH72
YP_003447322	*Azospirillum sp.* B510
YP_784834	*Bordetella avium* 197N
NP_886932.1	*Bordetella bronchiseptica* RB50
NP_882735.1	*Bordetella parapertussis* 12822
NP_879514.1	*Bordetella pertussis* Tohama I
YP_001633161.1	*Bordetella petrii* DSM 12804
YP_001807154.1	*Burkholderia ambifaria* MC40-6
NP_899749.1	*Chromobacterium violaceum* ATCC 12472
YP_003276820.1	*Comamonas testosteroni* CNB-2
YP_286888.1	*Dechloromonas aromatica* RCB
YP_003848303.1	*Gallionella capsiferriformans* ES-2
YP_001098549.1	*Herminiimonas arsenicoxydans*
YP_001351904.1	*Janthinobacterium sp.* Marseille
YP_002797094.1	*Laribacter hongkongensis* HLHK9
YP_420980	*Magnetospirillum magneticum* AMB-1
YP_001019721.1	*Methylibium petroleiphilum* PM1
YP_546579.1	*Methylobacillus flagellatus* KT
YP_003049669.1	*Methylotenera mobilis* JLW8
YP_003052281.1	*Methylovorus sp.* SIP3-4
NP_273620.1	*Neisseria meningitidis* MC58
NP_841936.1	*Nitrosomonas europaea* ATCC 19718
YP_747055.1	*Nitrosomonas eutropha* C91
YP_413321.1	*Nitrosospira multiformis* ATCC 25196
YP_001797072.1	*Polynucleobacter necessarius necessarius* STIR1
YP_001350647.1	*Pseudomonas aeruginosa* PA7
YP_727776.1	*Ralstonia eutropha* H16
YP_522546.1	*Rhodoferax ferrireducens* T118
YP_425840	*Rhodospirillum rubrum* ATCC 11170
YP_003522884.1	*Sideroxydans lithotrophicus* ES-1
YP_316250.1	*Thiobacillus denitrificans* ATCC 25259
NP_299925.1	*Xylella fastidiosa* 9a5c
YP_001162473.1	*Yersinia pestis* Pestoides F

GenBank accession numbers in bold typeface were sequenced in this work.
