# Supplementary material for: Identification and Phylogenetic Analysis of Heme Synthesis Genes in Trypanosomatids and Their Bacterial Endosymbionts
Source: PLoS One. 2011 Aug 10;6(8):e23518. doi: 10.1371/journal.pone.0023518 (PMC3154472; doi:10.1371/journal.pone.0023518)
Supplement: Table S3 — Proteins utilized in the phylogenetic analysis of glutamate-1-semialdehyde 2,1-aminomutase (GSA) and the respective organism names. (PDF) [file pone.0023518.s016.pdf]

Accession number	Organism
**JF756621**	*Candidatus* Kinetoplastibacterium blastocrithidii
**JF756622**	*Candidatus* Kinetoplastibacterium crithidii
**JF756623**	*Candidatus* Kinetoplastibacterium galatii
**JF756624**	*Candidatus* Kinetoplastibacterium oncopeltii
ZP_06685671.1	*Achromobacter piechaudii* ATCC 43553
YP_003976673	*Achromobacter xylosoxidans* A8
YP_157966.1	*Aromatoleum aromaticum* EbN1
YP_934951.1	*Azoarcus sp.* BH72
YP_787511	*Bordetella avium* 197N
NP_890943.1	*Bordetella bronchiseptica* RB50
NP_886086.1	*Bordetella parapertussis* 12822
NP_879190.1	*Bordetella pertussis* Tohama I
YP_001629160.1	*Bordetella petrii* DSM 12804
YP_001807540.1	*Burkholderia ambifaria* MC40-6
NP_899737.1	*Chromobacterium violaceum* ATCC 12472
YP_003280034.1	*Comamonas testosteroni* CNB-2
YP_287100.1	*Dechloromonas aromatica* RCB
YP_003845962.1	*Gallionella capsiferriformans* ES-2
YP_001100988.1	*Herminiimonas arsenicoxydans*
YP_001354649.1	*Janthinobacterium sp.* Marseille
YP_002794024.1	*Laribacter hongkongensis* HLHK9
YP_001022381.1	*Methylibium petroleiphilum* PM1
YP_546768.1	*Methylobacillus flagellatus* KT
YP_003049739.1	*Methylotenera mobilis* JLW8
YP_003052566.1	*Methylovorus sp.* SIP3-4
NP_274860.1	*Neisseria meningitidis* MC58
NP_841464.1	*Nitrosomonas europaea* ATCC 19718
YP_747746.1	*Nitrosomonas eutropha* C91
YP_413308.1	*Nitrosospira multiformis* ATCC 25196
YP_001797170.1	*Polynucleobacter necessarius necessarius* STIR1
YP_001346517.1	*Pseudomonas aeruginosa* PA7
YP_725250.1	*Ralstonia eutropha* H16
YP_522634.1	*Rhodoferax ferrireducens* T118
YP_003522668.1	*Sideroxydans lithotrophicus* ES-1
YP_316315.1	*Thiobacillus denitrificans* ATCC 25259
NP_299581.1	*Xylella fastidiosa* 9a5c
YP_001164301.1	*Yersinia pestis* Pestoides F

GenBank accession numbers in bold typeface were sequenced in this work.
