# Supplementary material for: Identification and Phylogenetic Analysis of Heme Synthesis Genes in Trypanosomatids and Their Bacterial Endosymbionts
Source: PLoS One. 2011 Aug 10;6(8):e23518. doi: 10.1371/journal.pone.0023518 (PMC3154472; doi:10.1371/journal.pone.0023518)
Supplement: Table S4 — Proteins utilized in the phylogenetic analysis of aminolevulinic acid synthase (ALAD) and the respective organism names. (PDF) [file pone.0023518.s017.pdf]

Accession number	Organism
**JF756617**	*Candidatus* Kinetoplastibacterium blastocrithidii
**JF756618**	*Candidatus* Kinetoplastibacterium crithidii
**JF756619**	*Candidatus* Kinetoplastibacterium galatii
**JF756620**	*Candidatus* Kinetoplastibacterium oncopeltii
ZP_06685034.1	*Achromobacter piechaudii* ATCC 43553
YP_003982416	*Achromobacter xylosoxidans* A8
YP_002549156.1	*Agrobacterium vitis* S4
YP_157589.1	*Aromatoleum aromaticum* EbN1
YP_934324.1	*Azoarcus sp.* BH72
YP_784603	*Bordetella avium* 197N
NP_886616.1	*Bordetella bronchiseptica* RB50
NP_882427.1	*Bordetella parapertussis* 12822
NP_882156.1	*Bordetella pertussis* Tohama I
YP_001633538.1	*Bordetella petrii* DSM 12804
YP_001807023.1	*Burkholderia ambifaria* MC40-6
YP_002516782	*Caulobacter crescentus* NA1000
NP_901318.1	*Chromobacterium violaceum* ATCC 12472
YP_003276336.1	*Comamonas testosteroni* CNB-2
YP_283853.1	*Dechloromonas aromatica* RCB
YP_003845964.1	*Gallionella capsiferriformans* ES-2
YP_001101366.1	*Herminiimonas arsenicoxydans*
YP_001355068.1	*Janthinobacterium sp.* Marseille
YP_002796882.1	*Laribacter hongkongensis* HLHK9
YP_001019503.1	*Methylibium petroleiphilum* PM1
YP_546570.1	*Methylobacillus flagellatus* KT
YP_003049605.1	*Methylotenera mobilis* JLW8
YP_003052273.1	*Methylovorus sp.* SIP3-4
NP_273843.1	*Neisseria meningitidis* MC58
NP_842448.1	*Nitrosomonas europaea* ATCC 19718
YP_746484.1	*Nitrosomonas eutropha* C91
YP_412765.1	*Nitrosospira multiformis* ATCC 25196
YP_001797022.1	*Polynucleobacter necessarius necessarius* STIR1
YP_001351303.1	*Pseudomonas aeruginosa* PA7
YP_727889.1	*Ralstonia eutropha* H16
YP_002525473	*Rhodobacter sphaeroides* KD131
YP_525036.1	*Rhodoferax ferrireducens* T118
YP_003522666.1	*Sideroxydans lithotrophicus* ES-1
YP_313947.1	*Thiobacillus denitrificans* ATCC 25259
NP_299585.1	*Xylella fastidiosa* 9a5c
YP_001164717.1	*Yersinia pestis* Pestoides F

GenBank accession numbers in bold typeface were sequenced in this work.
