# Supplementary material for: Identification and Phylogenetic Analysis of Heme Synthesis Genes in Trypanosomatids and Their Bacterial Endosymbionts
Source: PLoS One. 2011 Aug 10;6(8):e23518. doi: 10.1371/journal.pone.0023518 (PMC3154472; doi:10.1371/journal.pone.0023518)
Supplement: Table S5 — Proteins utilized in the phylogenetic analysis of porphobilinogen deaminase (PBGD) and the respective organism names. (PDF) [file pone.0023518.s018.pdf]

**Accession number**	**Organism**
**JF756613**	*Candidatus* Kinetoplastibacterium blastocrithidii
**JF756614**	*Candidatus* Kinetoplastibacterium crithidii
**JF756615**	*Candidatus* Kinetoplastibacterium galatii
**JF756616**	*Candidatus* Kinetoplastibacterium oncopeltii
ZP_06686723.1	*Achromobacter piechaudii* ATCC 43553
YP_003977929	*Achromobacter xylosoxidans* A8
YP_002551012	*Agrobacterium vitis* S4
YP_157636.1	*Aromatoleum aromaticum* EbN1
YP_932497.1	*Azoarcus sp.* BH72
YP_786814	*Bordetella avium* 197N
NP_888628.1	*Bordetella bronchiseptica* RB50
NP_884865.1	*Bordetella parapertussis* 12822
NP_881164.1	*Bordetella pertussis* Tohama I
YP_001630674.1	*Bordetella petrii* DSM 12804
YP_001809028.1	*Burkholderia ambifaria* MC40-6
YP_002515445	*Caulobacter crescentus* NA1000
NP_899724.2	*Chromobacterium violaceum* ATCC 12472
YP_003279574.1	*Comamonas testosteroni* CNB-2
YP_286873.1	*Dechloromonas aromatica* RCB
YP_003848274.1	*Gallionella capsiferriformans* ES-2
YP_001099316.1	*Herminiimonas arsenicoxydans*
YP_001352832.1	*Janthinobacterium sp.* Marseille
YP_002797123.1	*Laribacter hongkongensis* HLHK9
YP_001022190.1	*Methylibium petroleiphilum* PM1
YP_544147.1	*Methylobacillus flagellatus* KT
YP_003047603.1	*Methylotenera mobilis* JLW8
YP_003049810.1	*Methylovorus sp.* SIP3-4
NP_273584.1	*Neisseria meningitidis* MC58
NP_840674.1	*Nitrosomonas europaea* ATCC 19718
YP_747258.2	*Nitrosomonas eutropha* C91
YP_413369.1	*Nitrosospira multiformis* ATCC 25196
YP_001797959.1	*Polynucleobacter necessarius necessarius* STIR1
YP_001351322.1	*Pseudomonas aeruginosa* PA7
YP_727364.1	*Ralstonia eutropha* H16
YP_002526398	*Rhodobacter sphaeroides* KD131
YP_522975.1	*Rhodoferax ferrireducens* T118
YP_003522896.1	*Sideroxydans lithotrophicus* ES-1
YP_316326.1	*Thiobacillus denitrificans* ATCC 25259
NP_298916.1	*Xylella fastidiosa* 9a5c
YP_001164793.1	*Yersinia pestis* Pestoides F

GenBank accession numbers in bold typeface were sequenced in this work.
