# Supplementary material for: Identification and Phylogenetic Analysis of Heme Synthesis Genes in Trypanosomatids and Their Bacterial Endosymbionts
Source: PLoS One. 2011 Aug 10;6(8):e23518. doi: 10.1371/journal.pone.0023518 (PMC3154472; doi:10.1371/journal.pone.0023518)
Supplement: Table S6 — Proteins utilized in the phylogenetic analysis of uroporphyrinogen III synthase (UROS) and the respective organism names. (PDF) [file pone.0023518.s019.pdf]

Accession number	Organism
**JF756609**	*Candidatus* Kinetoplastibacterium blastocrithidii
**JF756610**	*Candidatus* Kinetoplastibacterium crithidii
**JF756611**	*Candidatus* Kinetoplastibacterium galatii
**JF756612**	*Candidatus* Kinetoplastibacterium oncopeltii
ZP_06686724.1	*Achromobacter piechaudii* ATCC 43553
YP_003977930	*Achromobacter xylosoxidans* A8
YP_002551013.1	*Agrobacterium vitis* S4
YP_157635.1	*Aromatoleum aromaticum* EbN1
YP_932498.1	*Azoarcus sp.* BH72
YP_786813	*Bordetella avium* 197N
NP_888629.1	*Bordetella bronchiseptica* RB50
NP_884866.1	*Bordetella parapertussis* 12822
NP_881163.1	*Bordetella pertussis* Tohama I
YP_001630675.1	*Bordetella petrii* DSM 12804
YP_001809027.1	*Burkholderia ambifaria* MC40-6
YP_002515446	*Caulobacter crescentus* NA1000
NP_899722.1	*Chromobacterium violaceum* ATCC 12472
YP_003279575.1	*Comamonas testosteroni* CNB-2
YP_286872.1	*Dechloromonas aromatica* RCB
YP_003848273.1	*Gallionella capsiferriformans* ES-2
YP_001099317.1	*Herminiimonas arsenicoxydans*
YP_001352833.1	*Janthinobacterium sp.* Marseille
YP_002794076.1	*Laribacter hongkongensis* HLHK9
YP_001022189	*Methylibium petroleiphilum* PM1
YP_544146	*Methylobacillus flagellatus* KT
YP_003047602.1	*Methylotenera mobilis* JLW8
YP_003049809.1	*Methylovorus sp.* SIP3-4
NP_273819.1	*Neisseria meningitidis* MC58
NP_840675.1	*Nitrosomonas europaea* ATCC 19718
YP_747259.1	*Nitrosomonas eutropha* C91
YP_413368.1	*Nitrosospira multiformis* ATCC 25196
YP_001797958.1	*Polynucleobacter necessarius necessarius* STIR1
YP_001351321.1	*Pseudomonas aeruginosa* PA7
YP_727363.1	*Ralstonia eutropha* H16
YP_002527262	*Rhodobacter sphaeroides* KD131
YP_522976.1	*Rhodoferax ferrireducens* T118
YP_003522897.1	*Sideroxydans lithotrophicus* ES-1
YP_316325.1	*Thiobacillus denitrificans* ATCC 25259
NP_299087	*Xylella fastidiosa* 9a5c
YP_001164794.1	*Yersinia pestis* Pestoides F

GenBank accession numbers in bold typeface were sequenced in this work.
