# Supplementary material for: Identification and Phylogenetic Analysis of Heme Synthesis Genes in Trypanosomatids and Their Bacterial Endosymbionts
Source: PLoS One. 2011 Aug 10;6(8):e23518. doi: 10.1371/journal.pone.0023518 (PMC3154472; doi:10.1371/journal.pone.0023518)
Supplement: Table S7 — Proteins utilized in the phylogenetic analysis of uroporphyrinogen III decarboxilase (UROD) and the respective organism names. (PDF) [file pone.0023518.s020.pdf]

Accession number	Organism
**JF756605**	*Candidatus* Kinetoplastibacterium blastocrithidii
**JF756606**	*Candidatus* Kinetoplastibacterium crithidii
**JF756607**	*Candidatus* Kinetoplastibacterium galatii
**JF756608**	*Candidatus* Kinetoplastibacterium oncopeltii
ZP_06685517.1	*Achromobacter piechaudii* ATCC 43553
YP_003976454	*Achromobacter xylosoxidans* A8
YP_002551206	*Agrobacterium vitis* S4
YP_158609.1	*Aromatoleum aromaticum* EbN1
YP_935481.1	*Azoarcus sp.* BH72
YP_787706	*Bordetella avium* 197N
NP_891134.1	*Bordetella bronchiseptica* RB50
NP_886266.1	*Bordetella parapertussis* 12822
NP_881833.1	*Bordetella pertussis* Tohama I
YP_001628949.1	*Bordetella petrii* DSM 12804
YP_001806828.1	*Burkholderia ambifaria* MC40-6
YP_002519252	*Caulobacter crescentus* NA1000
NP_900792.1	*Chromobacterium violaceum* ATCC 12472
YP_003276450.1	*Comamonas testosteroni* CNB-2
YP_283261.1	*Dechloromonas aromatica* RCB
YP_003845985.1	*Gallionella capsiferriformans* ES-2
YP_001101608.1	*Herminiimonas arsenicoxydans*
YP_001355297.1	*Janthinobacterium sp.* Marseille
YP_002796809.1	*Laribacter hongkongensis* HLHK9
YP_001019331.1	*Methylibium petroleiphilum* PM1
YP_546770.1	*Methylobacillus flagellatus* KT
YP_003049736.1	*Methylotenera mobilis* JLW8
YP_003052569.1	*Methylovorus sp.* SIP3-4
NP_273823.1	*Neisseria meningitidis* MC58
NP_840532.1	*Nitrosomonas europaea* ATCC 19718
YP_746840.1	*Nitrosomonas eutropha* C91
YP_411404.1	*Nitrosospira multiformis* ATCC 25196
YP_001796968.1	*Polynucleobacter necessarius necessarius* STIR1
YP_001351090.1	*Pseudomonas aeruginosa* PA7
YP_728058.1	*Ralstonia eutropha* H16
YP_002526399	*Rhodobacter sphaeroides* KD131
YP_522102.1	*Rhodoferax ferrireducens* T118
YP_003522746.1	*Sideroxydans lithotrophicus* ES-1
YP_316537.1	*Thiobacillus denitrificans* ATCC 25259
NP_298621.1	*Xylella fastidiosa* 9a5c
YP_001165055.1	*Yersinia pestis* Pestoides F

GenBank accession numbers in bold typeface were sequenced in this work.
