# Supplementary material for: Identification and Phylogenetic Analysis of Heme Synthesis Genes in Trypanosomatids and Their Bacterial Endosymbionts
Source: PLoS One. 2011 Aug 10;6(8):e23518. doi: 10.1371/journal.pone.0023518 (PMC3154472; doi:10.1371/journal.pone.0023518)
Supplement: Table S8 — Proteins utilized in the phylogenetic analysis of oxygen-independent coproporphyrinogen III oxidase (hemN) and the respective organism names. (PDF) [file pone.0023518.s021.pdf]

Accession number	Organism
**JF756601**	*Candidatus* Kinetoplastibacterium blastocrithidii
**JF756602**	*Candidatus* Kinetoplastibacterium crithidii
**JF756603**	*Candidatus* Kinetoplastibacterium galatii
**JF756604**	*Candidatus* Kinetoplastibacterium oncopeltii
ZP_06688009.1	*Achromobacter piechaudii* ATCC 43553
YP_002548276	*Agrobacterium vitis* S4
YP_158319.1	*Aromatoleum aromaticum* EbN1
YP_935467.1	*Azoarcus sp.* BH72
YP_786449	*Bordetella avium* 197N
NP_889491.1	*Bordetella bronchiseptica* RB50
NP_885176.1	*Bordetella parapertussis* 12822
NP_880328.1	*Bordetella pertussis* Tohama I
YP_001630884.1	*Bordetella petrii* DSM 12804
YP_001807574.1	*Burkholderia ambifaria* MC40-6
YP_002515519	*Caulobacter crescentus* NA1000
NP_900597.1	*Chromobacterium violaceum* ATCC 12472
YP_003279827.1	*Comamonas testosteroni* CNB-2
YP_287051.1	*Dechloromonas aromatica* RCB
YP_003846203.1	*Gallionella capsiferriformans* ES-2
YP_001100384.1	*Herminiimonas arsenicoxydans*
YP_001353024.1	*Janthinobacterium sp.* Marseille
YP_002795933.1	*Laribacter hongkongensis* HLHK9
YP_001021892.1	*Methylibium petroleiphilum* PM1
YP_544152.1	*Methylobacillus flagellatus* KT
YP_003047608.1	*Methylotenera mobilis* JLW8
YP_003049820.1	*Methylovorus sp.* SIP3-4
NP_273707.1	*Neisseria meningitidis* MC58
NP_840367.1	*Nitrosomonas europaea* ATCC 19718
YP_746559.1	*Nitrosomonas eutropha* C91
YP_410769.1	*Nitrosospira multiformis* ATCC 25196
YP_001797613.1	*Polynucleobacter necessarius necessarius* STIR1
YP_001345880.1	*Pseudomonas aeruginosa* PA7
YP_725460.1	*Ralstonia eutropha* H16
YP_522892.1	*Rhodoferax ferrireducens* T118
YP_003523033.1	*Sideroxydans lithotrophicus* ES-1
YP_314237.1	*Thiobacillus denitrificans* ATCC 25259
NP_298796.1	*Xylella fastidiosa* 9a5c
YP_001161952.1	*Yersinia pestis* Pestoides F

GenBank accession numbers in bold typeface were sequenced in this work.
