# Supplementary material for: Identification and Phylogenetic Analysis of Heme Synthesis Genes in Trypanosomatids and Their Bacterial Endosymbionts
Source: PLoS One. 2011 Aug 10;6(8):e23518. doi: 10.1371/journal.pone.0023518 (PMC3154472; doi:10.1371/journal.pone.0023518)
Supplement: Table S9 — Proteins utilized in the phylogenetic analysis of coproporphyrinogen III oxidase (CPOX) and the respective organism names. (PDF) [file pone.0023518.s022.pdf]

Accession number	Organism
**JF756596**	*Candidatus* Kinetoplastibacterium crithidii
**JF756665**	*Angomonas deanei* TCC036E
**JF756666**	*Angomonas deanei* TCC036E
scf7180000114303:24690..23785	*Crithidia fasciculata* Cf-C1
**JF756661**	*Endotrypanum schaudinni* TCC224
XP_001562058.1	*Leishmania braziliensis*
3EJO_A	*Leishmania donovani*
XP_001463209.1	*Leishmania infantum*
XP_001680900.1	*Leishmania major* Friedlin
197725360	*Leishmania naiffi*
**JF756659**	*Leptomonas costaricensis* TCC169E
**JF756660**	*Leptomonas costaricensis* TCC169E
**JF756662**	*Strigomonas culicis* TCC012E
**JF756663**	*Strigomonas galati* TCC219
**JF756664**	*Strigomonas oncopelti* TCC290E
YP_001514978.1	*Acaryochloris marina* MBIC11017
YP_003187334.1	*Acetobacter pasteurianus* IFO 3283-01
ZP_06686877.1	*Achromobacter piechaudii* ATCC 43553
YP_003978147.1	*Achromobacter xylosoxidans* A8
YP_001236053.1	*Acidiphilium cryptum* JF-5
ZP_05292918.1	*Acidithiobacillus caldus* ATCC 51756
YP_002220338.1	*Acidithiobacillus ferrooxidans* ATCC 53993
ZP_06212639.1	*Acidovorax avenae avenae* ATCC 19860
ZP_04763980.1	*Acidovorax delafieldii* 2AN
YP_002553162.1	*Acidovorax ebreus* TPSY
ZP_06066830.1	*Acinetobacter junii* SH205
ZP_06070563.1	*Acinetobacter lwoffii* SH145
XP_001952689.1	*Acyrthosiphon pisum*
XP_001663577.1	*Aedes aegypti*
YP_854793.1	*Aeromonas hydrophila hydrophila* ATCC 7966
YP_001143820.1	*Aeromonas salmonicida salmonicida* A449
ZP_07027835.1	*Afipia sp.* 1NLS2
YP_002545149.1	*Agrobacterium radiobacter* K84
NP_355204.1	*Agrobacterium tumefaciens* str. C58
YP_002550317.1	*Agrobacterium vitis* S4
ZP_07375603.1	*Ahrensia sp.* R2A130
EFB22733.1	*Ailuropoda melanoleuca*
EER37262.1	*Ajellomyces capsulatus* H143
EEQ90579.1	*Ajellomyces dermatitidis* ER-3
YP_691855.1	*Alcanivorax borkumensis* SK2
ZP_07720312.1	*Algoriphagus sp.* PR1
ZP_07021521.1	*Alicycliphilus denitrificans* BC
YP_743464.1	*Alkalilimnicola ehrlichii* MLHE-1
YP_003444553.1	*Allochromatium vinosum* DSM 180
ZP_02189150.1	alpha proteobacterium BAL199
ZP_06054942.1	alpha proteobacterium HIMB114
ZP_01446499.1	alpha proteobacterium HTCC2255
ZP_01614072.1	Alteromonadales bacterium TW-7
YP_002124342.1	*Alteromonas macleodii* 'Deep ecotype'
BAB41185.1	*Amaranthus tricolor*
YP_003764985.1	*Amycolatopsis mediterranei* U32

YP_325488.1	*Anabaena variabilis* ATCC 29413
YP_325073.1	*Anabaena variabilis* ATCC 29413
YP_002492357.1	*Anaeromyxobacter dehalogenans* 2CP-1
YP_001378891.1	*Anaeromyxobacter sp.* Fw109-5
YP_003328486.1	*Anaplasma centrale* str. Israel
YP_505026.1	*Anaplasma phagocytophilum* HZ
XP_001689093.1	*Anopheles gambiae* str. PEST
XP_624465.1	*Apis mellifera*
AAP34327.1	*Aplysia californica*
XP_002874876.1	*Arabidopsis lyrata lyrata*
YP_157632.1	*Aromatoleum aromaticum* EbN1
CBA72044.1	*Arsenophonus nasoniae*
XP_003017345.1	*Arthroderma benhamiae* CBS 112371
EFR03369.1	*Arthroderma gypseum* CBS 118893
XP_002845443.1	*Arthroderma otae* CBS 113480
ZP_03271485.1	*Arthrospira maxima* CS-328
NP_986695.1	*Ashbya gossypii* ATCC 10895
XP_001269417.1	*Aspergillus clavatus* NRRL 1
XP_750494.1	*Aspergillus fumigatus* Af293
CBF80916.1	*Aspergillus nidulans* FGSC A4
XP_001392079.1	*Aspergillus niger*
XP_001727930.1	*Aspergillus oryzae* RIB40
XP_001209481.1	*Aspergillus terreus* NIH2624
ZP_04771357.1	*Asticcacaulis excentricus* CB 48
ZP_01226211.1	*Aurantimonas manganoxydans* SI85-9A1
YP_934399.1	*Azoarcus sp.* BH72
YP_001526419.1	*Azorhizobium caulinodans* ORS 571
YP_003452324.1	*Azospirillum sp.* B510
YP_002797265.1	*Azotobacter vinelandii* DJ
CBW26108.1	*Bacteriovorax marinus* SJ
ZP_02000362.1	*Beggiatoa sp.* PS
YP_001831208.1	*Beijerinckia indica indica* ATCC 9039
NP_001040239.1	*Bombyx mori*
YP_786724.1	*Bordetella avium* 197N
NP_884655.1	*Bordetella parapertussis* 12822
NP_880948.1	*Bordetella pertussis* Tohama I
YP_001631711.1	*Bordetella petrii* DSM 12804
NP_001179322.1	*Bos taurus*
NP_769121.1	*Bradyrhizobium japonicum* USDA 110
YP_001238378.1	*Bradyrhizobium sp.* BTAi1
YP_001204071.1	*Bradyrhizobium sp.* ORS278
ZP_05033661.1	*Brevundimonas sp.* BAL3
YP_003819662.1	*Brevundimonas subvibrioides* ATCC 15264
YP_001628208.1	*Brucella suis* ATCC 23445
YP_001808908.1	*Burkholderia ambifaria* MC40-6
YP_002912416.1	*Burkholderia glumae* BGR1
ZP_02882884.1	*Burkholderia graminis* C4D1M
YP_001858290.1	*Burkholderia phymatum* STM815
YP_004029856.1	*Burkholderia rhizoxinica* HKI 454
ZP_02387100.1	*Burkholderia thailandensis* Bt4
ZP_02378303.1	*Burkholderia ubonensis* Bu
XP_002758917.1	*Callithrix jacchus*
EFN65629.1	*Camponotus floridanus*

XP_711014.1	*Candida albicans* SC5314
XP_002419319.1	*Candida dubliniensis* CD36
XP_448741.1	*Candida glabrata* CBS 138
XP_002548314.1	*Candida tropicalis* MYA-3404
YP_003166069.1	*Candidatus* Accumulibacter phosphatis clade IIA str. UW-1
ABV27411.1	*Candidatus* Chloracidobacterium thermophilum
YP_002924060.1	*Candidatus* Hamiltonella defensa 5AT (*Acyrthosiphon pisum*)
YP_003065486.1	*Candidatus* Liberibacter asiaticus str. psy62
ADR52468.1	*Candidatus* Liberibacter solanacearum CLso-ZC1
ZP_05068735.1	*Candidatus* Pelagibacter sp. HTCC7211
YP_265529.1	*Candidatus* Pelagibacter ubique HTCC1062
YP_008974.1	*Candidatus* Protochlamydia amoebophila UWE25
YP_003552513.1	*Candidatus* Puniceispirillum marinum IMCC1322
ZP_07396073.1	*Candidatus* Regiella insecticola LSR1
YP_903722.1	*Candidatus* Ruthia magnifica str. Cm (*Calyptogena magnifica*)
YP_001219299.1	*Candidatus* Vesicomyosocius okutanii HA
XP_545070.2	*Canis familiaris*
ZP_05705596.1	*Cardiobacterium hominis* ATCC 15826
NP_419325.1	*Caulobacter crescentus* CB15
YP_003591524.1	*Caulobacter segnis* ATCC 21756
YP_001984040.1	*Cellvibrio japonicus* Ueda107
XP_001225650.1	*Chaetomium globosum* CBS 148.51
YP_003126108.1	*Chitinophaga pinensis* DSM 2588
XP_001701729.1	*Chlamydomonas reinhardtii*
XP_001701791.1	*Chlamydomonas reinhardtii*
EFN57674.1	*Chlorella variabilis*
EFN53355.1	*Chlorella variabilis*
YP_002462638.1	*Chloroflexus aggregans* DSM 9485
NP_900427.1	*Chromobacterium violaceum* ATCC 12472
YP_574908.1	*Chromohalobacter salexigens* DSM 3043
XP_002131844.1	*Ciona intestinalis*
ZP_05782630.1	*Citreicella sp.* SE45
YP_001451949.1	*Citrobacter koseri* ATCC BAA-895
YP_003365934.1	*Citrobacter rodentium* ICC168
ZP_04562911.1	*Citrobacter sp.* 30_2
ZP_06351912.1	*Citrobacter youngae* ATCC 29220
ZP_06861256.1	*Citromicrobium bathyomarinum* JL354
XP_002615716.1	*Clavispora lusitaniae* ATCC 42720
XP_001248739.1	*Coccidioides immitis* RS
YP_270654.1	*Colwellia psychrerythraea* 34H
YP_003278939.1	*Comamonas testosteroni* CNB-2
ZP_01103085.1	*Congregibacter litoralis* KT71
XP_001831724.1	*Coprinopsis cinerea* okayama7#130
YP_003547458.1	*Coraliomargarita akajimensis* DSM 45221
NP_820710.2	*Coxiella burnetii* RSA 493
YP_003715487.1	*Croceibacter atlanticus* HTCC2559
ZP_00515789.1	*Crocosphaera watsonii* WH 8501
YP_001436926.1	*Cronobacter sakazakii* ATCC BAA-894
XP_571169.1	*Cryptococcus neoformans* var. neoformans JEC21
XP_001847646.1	*Culex quinquefasciatus*
YP_582938.1	*Cupriavidus metallidurans* CH34
YP_002004893.1	*Cupriavidus taiwanensis*
CBA26360.1	*Curvibacter* putative symbiont of *Hydra magnipapillata*

ZP_05044031.1	*Cyanobium sp.* PCC 7001
ZP_01727119.1	*Cyanothece sp.* CCY0110
YP_002482619.1	*Cyanothece sp.* PCC 7425
YP_003887027.1	*Cyanothece sp.* PCC 7822
YP_002371374.1	*Cyanothece sp.* PCC 8801
ZP_06309389.1	*Cylindrospermopsis raciborskii* CS-505
YP_678526.1	*Cytophaga hutchinsonii* ATCC 33406
CAK04614.2	*Danio rerio*
XP_461074.1	*Debaryomyces hansenii* CBS767
YP_286868.1	*Dechloromonas aromatica* RCB
YP_001564544.1	*Delftia acidovorans* SPH-1
YP_003332374.1	*Dickeya dadantii* Ech586
YP_003005588.1	*Dickeya zeae* Ech1591
XP_636472.1	*Dictyostelium discoideum* AX4
YP_001532021.1	*Dinoroseobacter shibae* DFL 12
ZP_01049608.1	*Dokdonia donghaensis* MED134
XP_001962390.1	*Drosophila ananassae*
XP_001992983.1	*Drosophila grimshawi*
NP_524777.1	*Drosophila melanogaster*
XP_002003102.1	*Drosophila mojavensis*
XP_001355783.1	*Drosophila pseudoobscura pseudoobscura*
XP_002078453.1	*Drosophila simulans*
XP_002052468.1	*Drosophila virilis*
XP_002066428.1	*Drosophila willistoni*
YP_003086752.1	*Dyadobacter fermentans* DSM 18053
CBN73780.1	*Ectocarpus siliculosus*
CBJ29596.1	*Ectocarpus siliculosus*
ZP_06713957.1	*Edwardsiella tarda* ATCC 23685
YP_303084.1	*Ehrlichia canis* str. Jake
YP_507402.1	*Ehrlichia chaffeensis* str. Arkansas
YP_196393.1	*Ehrlichia ruminantium* str. Gardel
ZP_05619665.1	*Enhydrobacter aerosaccus* SK60
ZP_05968627.1	*Enterobacter cancerogenus* ATCC 35316
YP_003940871.1	*Enterobacter cloacae* SCF1
CAZ90522.1	*Enterobacter turicensis*
XP_001502354.2	*Equus caballus*
YP_003531857.1	*Erwinia amylovora* CFBP1430
YP_003742683.1	*Erwinia billingiae* Eb661
YP_001907028.1	*Erwinia tasmaniensis* Et1/99
YP_458773.1	*Erythrobacter litoralis* HTCC2594
ZP_01863945.1	*Erythrobacter sp.* SD-21
ZP_02901842.1	*Escherichia albertii* TW07627
YP_003911319.1	*Ferrimonas balearica* DSM 9799
ZP_01733825.1	Flavobacteria bacterium BAL38
ZP_01201439.1	Flavobacteria bacterium BBFL7
ZP_03702571.1	Flavobacteria bacterium MS024-2A
ZP_03700872.1	Flavobacteria bacterium MS024-3C
ZP_02181448.1	Flavobacteriales bacterium ALC-1
YP_003861950.1	Flavobacteriales bacterium HTCC2170
YP_001193303.1	*Flavobacterium johnsoniae* UW101
YP_001294977.1	*Flavobacterium psychrophilum* JIP02/86
ZP_04756187.1	*Francisella philomiragia philomiragia* ATCC 25015
YP_763529.1	*Francisella tularensis holarctica* OSU18

ZP_01437668.1	*Fulvimarina pelagi* HTCC2506
YP_003848417.1	*Gallionella capsiferriformans* ES-2
XP_416596.1	*Gallus gallus*
ZP_05128028.1	gamma proteobacterium NOR5-3
ZP_04956597.1	gamma proteobacterium NOR51-B
ZP_02736979.1	*Gemmata obscuriglobus* UQM 2246
YP_002760648.1	*Gemmatimonas aurantiaca* T-27
XP_390915.1	*Gibberella zeae* PH-1
ZP_03561580.1	*Glaciecola sp.* HTCC2999
NP_926822.1	*Gloeobacter violaceus* PCC 7421
EFQ32913.1	*Glomerella graminicola* M1.001
EFQ34236.1	*Glomerella graminicola* M1.001
ADD19859.1	*Glossina morsitans morsitans*
YP_001602303.1	*Gluconacetobacter diazotrophicus* PAI 5
ZP_06833757.1	*Gluconacetobacter hansenii* ATCC 23769
YP_192290.1	*Gluconobacter oxydans* 621H
P35055.1	*Glycine max*
YP_863238.1	*Gramella forsetii* KT0803
YP_745543.1	*Granulibacter bethesdensis* CGDNIH1
ZP_06050889.1	*Grimontia hollisae* CIP 101886
ABD51922.1	*Guillardia theta*
YP_431382.1	*Hahella chejuensis* KCTC 2396
YP_003896447.1	*Halomonas elongata* DSM 2581
YP_001003885.1	*Halorhodospira halophila* SL1
YP_003263957.1	*Halothiobacillus neapolitanus* c2
EFN81420.1	*Harpegnathos saltator*
YP_003776946.1	*Herbaspirillum seropedicae* SmR1
YP_001098889.1	*Herminiimonas arsenicoxydans*
YP_003059131.1	*Hirschia baltica* ATCC 49814
ZP_02168689.1	*Hoeflea phototrophica* DFL-43
BAA04033.1	*Homo sapiens*
Q42840.1	*Hordeum vulgare*
YP_003756649.1	*Hyphomicrobium denitrificans* ATCC 51888
YP_759079.1	*Hyphomonas neptunium* ATCC 15444
ZP_01042061.1	*Idiomarina baltica* OS145
YP_154416.1	*Idiomarina loihiensis* L2TR
ABA55505.1	*Isochrysis galbana*
XP_002406476.1	*Ixodes scapularis*
YP_511790.1	*Jannaschia sp.* CCS1
YP_001352232.1	*Janthinobacterium sp.* Marseille
YP_003147787.1	*Kangiella koreensis* DSM 16069
YP_003963359.1	*Ketogulonicigenium vulgare* Y25
YP_002237218.1	*Klebsiella pneumoniae* 342
XP_455911.1	*Kluyveromyces lactis* NRRL Y-1140
ZP_02161920.1	*Kordia algicida* OT-1
YP_003384119.1	*Kribbella flavida* DSM 17836
ZP_05115343.1	*Labrenzia alexandrii* DFL-11
XP_001883701.1	*Laccaria bicolor* S238N-H82
XP_002552538.1	*Lachancea thermotolerans*
YP_002794719.1	*Laribacter hongkongensis* HLHK9
YP_003996846.1	*Leadbetterella byssophila* DSM 17132
ZP_01059623.1	*Leeuwenhoekiella blandensis* MED217
YP_003454795.1	*Legionella longbeachae* NSW150

YP_123547.1	*Legionella pneumophila* str. Paris
ZP_01876308.1	*Lentisphaera araneosa* HTCC2155
YP_001790983.1	*Leptothrix cholodnii* SP-6
ZP_01914998.1	*Limnobacter sp.* MED105
XP_001527171.1	*Lodderomyces elongisporus* NRRL YB-4239
ZP_01003942.1	*Loktanella vestfoldensis* SKA53
ZP_03696669.1	*Lutiella nitroferrum* 2002
ZP_01620260.1	*Lyngbya sp.* PCC 8106
XP_001088605.1	*Macaca mulatta*
XP_368183.2	*Magnaporthe oryzae* 70-15
ZP_00052048.1	*Magnetospirillum magnetotacticum* MS-1
XP_001731979.1	*Malassezia globosa* CBS 7966
YP_755965.1	*Maricaulis maris* MCS10
ZP_01625673.1	marine gamma proteobacterium HTCC2080
ZP_01615626.1	marine gamma proteobacterium HTCC2143
ZP_05096563.1	marine gamma proteobacterium HTCC2148
ZP_01223348.1	marine gamma proteobacterium HTCC2207
ZP_01893383.1	*Marinobacter algicola* DG893
YP_957341.1	*Marinobacter aquaeolei* VT8
ZP_01076872.1	*Marinomonas sp.* MED121
YP_001338938.1	*Marinomonas sp.* MWYL1
ZP_01013122.1	*Maritimibacter alkaliphilus* HTCC2654
YP_004053588.1	*Marivirga tractuosa* DSM 4126
NP_103997.1	*Mesorhizobium loti* MAFF303099
ZP_05810785.1	*Mesorhizobium opportunistum* WSM2075
YP_674726.1	*Mesorhizobium sp.* BNC1
YP_001020536.1	*Methylibium petroleiphilum* PM1
YP_544458.1	*Methylobacillus flagellatus* KT
ZP_07652679.1	*Methylobacter tundripaludum* SV96
YP_001638156.1	*Methylobacterium extorquens* PA1
YP_002501670.1	*Methylobacterium nodulans* ORS 2060
YP_001757320.1	*Methylobacterium radiotolerans* JCM 2831
YP_002364048.1	*Methylocella silvestris* BL2
YP_115186.1	*Methylococcus capsulatus* str. Bath
ZP_05104306.1	*Methylophaga thiooxidans* DMS010
ZP_01552641.1	*Methylophilales bacterium* HTCC2181
YP_003047838.1	*Methylotenera mobilis* JLW8
YP_003673351.1	*Methylotenera sp.* 301
YP_003050153.1	*Methylovorus sp.* SIP3-4
ZP_05026772.1	*Microcoleus chthonoplastes* PCC 7420
ZP_05027767.1	*Microcoleus chthonoplastes* PCC 7420
YP_001655684.1	*Microcystis aeruginosa* NIES-843
XP_003062314.1	*Micromonas pusilla* CCMP1545
XP_003064620.1	*Micromonas pusilla* CCMP1545
ZP_01686958.1	*Microscilla marina* ATCC 23134
XP_001380630.1	*Monodelphis domestica*
XP_001749080.1	*Monosiga brevicollis* MX1
YP_003627566.1	*Moraxella catarrhalis* RH4
ZP_01900750.1	*Moritella sp.* PE36
ZP_07746427.1	*Mucilaginibacter paludis* DSM 18603
AAH17680.2	*Mus musculus*
YP_634879.1	*Myxococcus xanthus* DK 1622
XP_001603631.1	*Nasonia vitripennis*

XP_003048044.1	*Nectria haematococca* mpVI 77-13-4
ZP_06734703.1	*Neisseria elongata glycolytica* ATCC 29315
ZP_04758168.1	*Neisseria flavescens* SK114
ZP_05977631.1	*Neisseria mucosa* ATCC 25996
ZP_05319503.1	*Neisseria sicca* ATCC 29256
ZP_06980038.1	*Neisseria sp.* oral taxon 014 str. F0314
ZP_05985247.1	*Neisseria subflava* NJ9703
XP_001620068.1	*Nematostella vectensis*
YP_003081649.1	*Neorickettsia risticii* str. Illinois
YP_506339.1	*Neorickettsia sennetsu* str. Miyayama
XP_001264923.1	*Neosartorya fischeri* NRRL 181
XP_956249.2	*Neurospora crassa* OR74A
Q42946.1	*Nicotiana tabacum*
YP_578441.1	*Nitrobacter hamburgensis* X14
ZP_01045264.1	*Nitrobacter sp.* Nb-311A
YP_319226.1	*Nitrobacter winogradskyi* Nb-255
ZP_01126645.1	*Nitrococcus mobilis* Nb-231
YP_003528877.1	*Nitrosococcus halophilus* Nc4
YP_003759555.1	*Nitrosococcus watsoni* C-113
NP_841898.1	*Nitrosomonas europaea* ATCC 19718
YP_748151.1	*Nitrosomonas eutropha* C91
ZP_05316517.1	*Nitrosomonas sp.* AL212
YP_410836.1	*Nitrosospira multiformis* ATCC 25196
ZP_01629829.1	*Nodularia spumigena* CCY9414
ZP_01632122.1	*Nodularia spumigena* CCY9414
YP_003721629.1	*Nostoc azollae* 0708
YP_001868443.1	*Nostoc punctiforme* PCC 73102
NP_485400.1	*Nostoc sp.* PCC 7120
YP_497806.1	*Novosphingobium aromaticivorans* DSM 12444
ZP_02152526.1	*Oceanibulbus indolifex* HEL-45
ZP_00953108.1	*Oceanicaulis alexandrii* HTCC2633
ZP_00998616.1	*Oceanicola batsensis* HTCC2597
ZP_01157099.1	*Oceanicola granulosus* HTCC2516
ZP_01306458.1	*Oceanobacter sp.* RED65
ZP_01165249.1	*Oceanospirillum sp.* MED92
YP_001370161.1	*Ochrobactrum anthropi* ATCC 49188
ZP_05066868.1	*Octadecabacter antarcticus* 238
CBY19597.1	*Oikopleura dioica*
YP_002289973.1	*Oligotropha carboxidovorans* OM5
YP_001819577.1	*Opitutus terrae* PB90-1
YP_001938470.1	*Orientia tsutsugamushi* str. Ikeda
XP_001519105.1	*Ornithorhynchus anatinus*
XP_002716787.1	*Oryctolagus cuniculus*
EEE61660.1	*Oryza sativa* Japonica Group
ZP_07111030.1	*Oscillatoria sp.* PCC 6506
XP_001416682.1	*Ostreococcus lucimarinus* CCE9901
XP_001422270.1	*Ostreococcus lucimarinus* CCE9901
XP_003078228.1	*Ostreococcus tauri*
XP_516615.2	*Pan troglodytes*
YP_003521072.1	*Pantoea ananatis* LMG 20103
YP_003931839.1	*Pantoea vagans* C9-1
ZP_06298692.1	*Parachlamydia acanthamoebae* str. Hall's coccus
XP_002790127.1	*Paracoccidioides brasiliensis* Pb01

YP_915545.1	*Paracoccus denitrificans* PD1222
XP_001449237.1	*Paramecium tetraurelia* strain d4-2
YP_001413511.1	*Parvibaculum lavamentivorans* DS-1
YP_003855304.1	*Parvularcula bermudensis* HTCC2503
YP_002049255.1	*Paulinella chromatophora*
YP_003258417.1	*Pectobacterium wasabiae* WPP163
XP_002425624.1	*Pediculus humanus corporis*
YP_003092428.1	*Pedobacter heparinus* DSM 2366
EFR81638.1	*Pedobacter saltans* DSM 12145
ZP_01882631.1	*Pedobacter sp.* BAL39
ZP_01444939.1	*Pelagibaca bermudensis* HTCC2601
XP_002557383.1	*Penicillium chrysogenum* Wisconsin 54-1255
XP_002149185.1	*Penicillium marneffei* ATCC 18224
ZP_02144631.1	*Phaeobacter gallaeciensis* BS107
XP_002186510.1	*Phaeodactylum tricornutum* CCAP 1055/1
XP_002182874.1	*Phaeodactylum tricornutum* CCAP 1055/1
XP_002179603.1	*Phaeodactylum tricornutum* CCAP 1055/1
XP_001791922.1	*Phaeosphaeria nodorum* SN15
YP_002131712.1	*Phenylobacterium zucineum* HLK1
ZP_06156466.1	*Photobacterium damselae damselae* CIP 102761
YP_131630.1	*Photobacterium profundum* SS9
ZP_01161226.1	*Photobacterium sp.* SKA34
YP_003040230.1	*Photorhabdus asymbiotica*
NP_928683.1	*Photorhabdus luminescens laumondii* TTO1
XP_001754127.1	*Physcomitrella patens patens*
XP_001766305.1	*Physcomitrella patens patens*
XP_002997752.1	*Phytophthora infestans* T30-4
ABR16358.1	*Picea sitchensis*
ADE77306.1	*Picea sitchensis*
EDK40106.2	*Pichia guilliermondii* ATCC 6260
XP_002493182.1	*Pichia pastoris* GS115
XP_675246.1	*Plasmodium berghei* strain ANKA
AAZ08394.1	*Plasmodium falciparum*
XP_725970.1	*Plasmodium yoelii yoelii* str. 17XNL
XP_001909319.1	*Podospora anserina* S mat+
ZP_01117858.1	*Polaribacter irgensii* 23-P
ZP_01052456.1	*Polaribacter sp.* MED152
YP_981964.1	*Polaromonas naphthalenivorans* CJ2
YP_001155398.1	*Polynucleobacter necessarius asymbioticus* QLW-P1DMWA-1
YP_001797988.1	*Polynucleobacter necessarius necessarius* STIR1
EFA74914.1	*Polysphondylium pallidum* PN500
XP_002813417.1	*Pongo abelii*
EEF08708.1	*Populus trichocarpa*
XP_002317199.1	*Populus trichocarpa*
XP_002472829.1	*Postia placenta* Mad-698-R
YP_001015848.1	*Prochlorococcus marinus* str. NATL1A
YP_002151566.1	*Proteus mirabilis* HI4320
ZP_03803516.1	*Proteus penneri* ATCC 35198
ZP_03318736.1	*Providencia alcalifaciens* DSM 30120
YP_659616.1	*Pseudoalteromonas atlantica* T6c
YP_338586.1	*Pseudoalteromonas haloplanktis* TAC125
ZP_01135228.1	*Pseudoalteromonas tunicata* D2
YP_002437634.1	*Pseudomonas aeruginosa* LESB58

YP_605841.1	*Pseudomonas entomophila* L48
YP_002869720.1	*Pseudomonas fluorescens* SBW25
YP_001185566.1	*Pseudomonas mendocina* ymp
ADR57811.1	*Pseudomonas putida* BIRD-1
YP_001170576.1	*Pseudomonas stutzeri* A1501
NP_790029.1	*Pseudomonas syringae* pv. tomato str. DC3000
ZP_05083700.1	*Pseudovibrio sp.* JE062
YP_263526.1	*Psychrobacter arcticus* 273-4
YP_579511.1	*Psychrobacter cryohalolentis* K5
ZP_01252416.1	*Psychroflexus torquis* ATCC 700755
EFQ89747.1	*Pyrenophora teres* f. teres 0-1
XP_001934028.1	*Pyrenophora tritici-repentis* Pt-1C-BFP
YP_725428.1	*Ralstonia eutropha* H16
YP_296729.1	*Ralstonia eutropha* JMP134
YP_001899956.1	*Ralstonia pickettii* 12J
YP_003751885.1	*Ralstonia solanacearum* PSI07
ZP_06306274.1	*Raphidiopsis brookii* D9
NP_001032172.1	*Rattus norvegicus*
ZP_01116580.1	*Reinekea sp.* MED297
YP_001979332.1	*Rhizobium etli* CIAT 652
YP_002282282.1	*Rhizobium leguminosarum* bv. trifolii WSM2304
YP_002826197.1	*Rhizobium sp.* NGR234
ZP_05842205.1	*Rhodobacter sp.* SW2
YP_002526401.1	*Rhodobacter sphaeroides* KD131
ZP_05124303.1	Rhodobacteraceae bacterium KLH11
ZP_05076255.1	Rhodobacterales bacterium HTCC2083
ZP_01740835.1	Rhodobacterales bacterium HTCC2150
YP_523333.1	*Rhodoferax ferrireducens* T118
YP_004011658.1	*Rhodomicrobium vannielii* ATCC 17100
YP_001990704.1	*Rhodopseudomonas palustris* TIE-1
YP_002299590.1	*Rhodospirillum centenum* SW
YP_003290471.1	*Rhodothermus marinus* DSM 4252
XP_002518396.1	*Ricinus communis*
YP_538550.1	*Rickettsia bellii* RML369-C
ZP_04698378.1	*Rickettsia* endosymbiont of *Ixodes scapularis*
YP_247412.1	*Rickettsia felis* URRWXCal2
YP_067807.1	*Rickettsia typhi* str. Wilmington
ZP_02062580.1	*Rickettsiella grylli*
YP_003194207.1	*Robiginitalea biformata* HTCC2501
ZP_07659502.1	*Roseibium sp.* TrichSKD4
YP_681614.1	*Roseobacter denitrificans* OCh 114
ZP_02141385.1	*Roseobacter litoralis* Och 149
ZP_01902436.1	*Roseobacter sp.* AzwK-3b
ZP_01749205.1	*Roseobacter sp.* CCS2
ZP_05100664.1	*Roseobacter sp.* GAI101
ZP_01057676.1	*Roseobacter sp.* MED193
ZP_01756322.1	*Roseobacter sp.* SK209-2-6
ZP_06898807.1	*Roseomonas cervicalis* ATCC 49957
ZP_00959751.1	*Roseovarius nubinhibens* ISM
ZP_01878086.1	*Roseovarius sp.* TM1035
YP_168848.1	*Ruegeria pomeroyi* DSS-3
ZP_05087730.1	*Ruegeria sp.* R11
NP_010329.1	*Saccharomyces cerevisiae* S288c

YP_525501.1	*Saccharophagus degradans* 2-40
XP_002730678.1	*Saccoglossus kowalevskii*
ZP_01747867.1	*Sagittula stellata* E-37
YP_003571828.1	*Salinibacter ruber* M8
ACI33288.1	*Salmo salar*
YP_001569509.1	*Salmonella enterica arizonae* serovar 62:z4,z23:-- str. RSK2980
XP_001386432.1	*Scheffersomyces stipitis* CBS 6054
CAX72893.1	*Schistosoma japonicum*
XP_002578608.1	*Schistosoma mansoni*
XP_003032760.1	*Schizophyllum commune* H4-8
XP_002176014.1	*Schizosaccharomyces japonicus* yFS275
NP_593150.1	*Schizosaccharomyces pombe* 972h-
XP_001597409.1	*Sclerotinia sclerotiorum* 1980
XP_002990281.1	*Selaginella moellendorffii*
ZP_06191243.1	*Serratia odorifera* 4Rx13
YP_001479690.1	*Serratia proteamaculans* 568
YP_925932.1	*Shewanella amazonensis* SB2B
YP_001095854.1	*Shewanella loihica* PV-4
YP_404179.1	*Shigella dysenteriae* Sd197
YP_003522925.1	*Sideroxydans lithotrophicus* ES-1
ZP_05786416.1	*Silicibacter lacuscaerulensis* ITI-1157
ZP_05741168.1	*Silicibacter sp.* TrichCH4B
YP_001327226.1	*Sinorhizobium medicae* WSM419
NP_385933.1	*Sinorhizobium meliloti* 1021
YP_455392.1	*Sodalis glossinidius* str. 'morsitans'
CBI53059.1	*Sordaria macrospora*
XP_002447075.1	*Sorghum bicolor*
XP_002445217.1	*Sorghum bicolor*
ZP_03966656.1	*Sphingobacterium spiritivorum* ATCC 33300
ZP_07573676.1	*Sphingobium chlorophenolicum* L-1
YP_003546501.1	*Sphingobium japonicum* UT26S
YP_001261900.1	*Sphingomonas wittichii* RW1
YP_616394.1	*Sphingopyxis alaskensis* RB2256
YP_003387114.1	*Spirosoma linguale* DSM 74
ZP_01549351.1	*Stappia aggregata* IAM 12614
YP_003694395.1	*Starkeya novella* DSM 506
YP_001974217.1	*Stenotrophomonas maltophilia* K279a
ZP_01465041.1	*Stigmatella aurantiaca* DW4/3-1
XP_001191557.1	*Strongylocentrotus purpuratus*
ZP_00954066.1	*Sulfitobacter sp.* EE-36
XP_003132767.1	*Sus scrofa*
YP_171565.1	*Synechococcus elongatus* PCC 6301
YP_474336.1	*Synechococcus sp.* JA-3-3Ab
YP_001735072.1	*Synechococcus sp.* PCC 7002
ZP_05035281.1	*Synechococcus sp.* PCC 7335
YP_001224184.1	*Synechococcus sp.* WH 7803
NP_898131.1	*Synechococcus sp.* WH 8102
NP_440183.1	*Synechocystis sp.* PCC 6803
XP_002190683.1	*Taeniopygia guttata*
XP_002484998.1	*Talaromyces stipitatus* ATCC 10500
YP_003071738.1	*Teredinibacter turnerae* T7901
XP_001031962.1	*Tetrahymena thermophila*
CAG08052.1	*Tetraodon nigroviridis*

ZP_05341965.1	*Thalassiobium sp.* R2A62
ZP_05342454.1	*Thalassiobium sp.* R2A62
XP_002287097.1	*Thalassiosira pseudonana* CCMP1335
XP_002294357.1	*Thalassiosira pseudonana* CCMP1335
YP_002355096.1	*Thauera sp.* MZ1T
NP_681473.1	*Thermosynechococcus elongatus* BP-1
YP_003461505.1	*Thioalkalivibrio sp.* K90mix
YP_316219.1	*Thiobacillus denitrificans* ATCC 25259
YP_390288.1	*Thiomicrospira crunogena* XCL-2
YP_003644034.1	*Thiomonas intermedia* K12
XP_002370089.1	*Toxoplasma gondii* ME49
EEZ98060.1	*Tribolium castaneum*
ACB56643.1	*Trichoderma aureoviride*
YP_721000.1	*Trichodesmium erythraeum* IMS101
XP_003024735.1	*Trichophyton verrucosum* HKI 0517
XP_002111571.1	*Trichoplax adhaerens*
XP_002118953.1	*Trichoplax adhaerens*
YP_003703904.1	*Truepera radiovictrix* DSM 17093
XP_002836050.1	*Tuber melanosporum* Mel28
XP_002543691.1	*Uncinocarpus reesii* 1704
ADI17025.1	uncultured Vibrionales bacterium HF0010_22E23
ZP_01889302.1	unidentified eubacterium SCB49
XP_758464.1	*Ustilago maydis* 521
XP_001644528.1	*Vanderwaltozyma polyspora* DSM 70294
YP_002944576.1	*Variovorax paradoxus* S110
YP_998963.1	*Verminephrobacter eiseniae* EF01-2
ZP_05057531.1	Verrucomicrobiae bacterium DG1235
XP_003006045.1	*Verticillium albo-atrum* VaMs.102
ZP_01236983.1	*Vibrio angustum* S14
YP_002808843.1	*Vibrio cholerae* M66-2
ZP_05883922.1	*Vibrio coralliilyticus* ATCC BAA-450
ZP_05880024.1	*Vibrio furnissii* CIP 102972
ZP_06176981.1	*Vibrio harveyi* 1DA3
ZP_05883493.1	*Vibrio metschnikovii* CIP 69.14
ZP_05119392.1	*Vibrio parahaemolyticus* 16
ZP_01868698.1	*Vibrio shilonii* AK1
ZP_00992206.1	*Vibrio splendidus* 12B01
NP_760016.1	*Vibrio vulnificus* CMCP6
CAN76809.1	*Vitis vinifera*
XP_002947376.1	*Volvox carteri* f. nagariensis
XP_002946372.1	*Volvox carteri* f. nagariensis
YP_003710114.1	*Waddlia chondrophila* WSU 86-1044
NP_871587.1	*Wigglesworthia glossinidia* endosymbiont of *Glossina brevipalpis*
YP_001975717.1	*Wolbachia* endosymbiont of *Culex quinquefasciatus* Pel
ZP_00373119.1	*Wolbachia* endosymbiont of *Drosophila ananassae*
NP_966926.1	*Wolbachia* endosymbiont of *Drosophila melanogaster*
ZP_03788199.1	*Wolbachia* endosymbiont of *Muscidifurax uniraptor*
YP_198539.1	*Wolbachia* endosymbiont strain TRS of *Brugia malayi*
YP_001417847.1	*Xanthobacter autotrophicus* Py2
YP_003374652.1	*Xanthomonas albilineans*
NP_644408.1	*Xanthomonas axonopodis* pv. citri str. 306
YP_001905617.1	*Xanthomonas campestris* pv. campestris str. B100
ZP_06705382.1	*Xanthomonas fuscans aurantifolii* str. ICPB 11122

YP_202878.1	*Xanthomonas oryzae* pv. oryzae KACC10331
XP_002945148.1	*Xenopus (Silurana) tropicalis*
YP_003468888.1	*Xenorhabdus bovienii* SS-2004
YP_003713496.1	*Xenorhabdus nematophila* ATCC 19061
AAF82830.1	*Xylella fastidiosa* 9a5c
XP_504574.1	*Yarrowia lipolytica*
ZP_04620598.1	*Yersinia aldovae* ATCC 35236
ZP_04627904.1	*Yersinia bercovieri* ATCC 43970
YP_001005494.1	*Yersinia enterocolitica enterocolitica* 8081
ZP_04631885.1	*Yersinia frederiksenii* ATCC 33641
ZP_04635700.1	*Yersinia intermedia* ATCC 29909
ZP_04625682.1	*Yersinia kristensenii* ATCC 33638
ZP_04642652.1	*Yersinia mollaretii* ATCC 43969
NP_668771.1	*Yersinia pestis* KIM 10
YP_001400260.1	*Yersinia pseudotuberculosis* IP 31758
ZP_04613522.1	*Yersinia rohdei* ATCC 43380
ZP_04617979.1	*Yersinia ruckeri* ATCC 29473
NP_001182775.1	*Zea mays*
YP_003582666.1	*Zunongwangia profunda* SM-A87
XP_002496228.1	*Zygosaccharomyces rouxii*
ZP_04759917.1	*Zymomonas mobilis mobilis* ATCC 10988

GenBank accession numbers (except for *C. fasciculata*, which has the TriTrypDB scaffold number and coordinates) in bold typeface were sequenced in this work.
