# Supplementary material for: Identification and Phylogenetic Analysis of Heme Synthesis Genes in Trypanosomatids and Their Bacterial Endosymbionts
Source: PLoS One. 2011 Aug 10;6(8):e23518. doi: 10.1371/journal.pone.0023518 (PMC3154472; doi:10.1371/journal.pone.0023518)
Supplement: Table S10 — Proteins utilized in the phylogenetic analysis of protoporphyrinogen oxidase (PPOX) and the respective organism names. (PDF) [file pone.0023518.s023.pdf]

Accession number	Organism
**JF756595**	*Candidatus* Kinetoplastibacterium crithidii
**JF756594**	*Candidatus* Kinetoplastibacterium desouzaii
**JF756635**	*Angomonas deanei* TCC036E
**JF756634**	*Crithidia acanthocephali* TCC037E
scf7180000114303:25388..26083	*Crithidia fasciculata* Cf-C1
**JF756638**	*Endotrypanum schaudinni* TCC224
XP_001562059.1	*Leishmania braziliensis*
XP_001463210.1	*Leishmania infantum*
XP_001680901.1	*Leishmania major* Friedlin
**JF756639**	*Leptomonas costaricensis* TCC169E
**JF756633**	*Strigomonas culicis* TCC012E
**JF756640**	*Strigomonas galati* TCC219
**JF756641**	*Strigomonas galati* TCC219
**JF756642**	*Strigomonas galati* TCC219
**JF756643**	*Strigomonas galati* TCC219
**JF756644**	*Strigomonas galati* TCC219
**JF756645**	*Strigomonas galati* TCC219
**JF756646**	*Strigomonas galati* TCC219
**JF756647**	*Strigomonas galati* TCC219
**JF756648**	*Strigomonas galati* TCC219
**JF756636**	*Strigomonas oncopelti* TCC290E
**JF756637**	*Strigomonas oncopelti* TCC290E
ZP_05630057.1	*Actinobacillus minor* 202
YP_003255211.1	*Aggregatibacter actinomycetemcomitans* D11S-1
YP_002264302.1	*Aliivibrio salmonicida* LFI1238
CBA71816.1	*Arsenophonus nasoniae*
YP_002924509.1	*Candidatus* Hamiltonella defensa 5AT (*Acyrthosiphon pisum*)
ZP_07394781.1	*Candidatus* Regiella insecticola LSR1
YP_001451798.1	*Citrobacter koseri* ATCC BAA-895
YP_003367343.1	*Citrobacter rodentium* ICC168
ZP_04558445.1	*Citrobacter sp.* 30_2
ZP_06354352.1	*Citrobacter youngae* ATCC 29220
YP_001439743.1	*Cronobacter sakazakii* ATCC BAA-894
YP_003331806.1	*Dickeya dadantii* Ech586
YP_003006176.1	*Dickeya zeae* Ech1591
ZP_06716269.1	*Edwardsiella tarda* ATCC 23685
ZP_05970968.1	*Enterobacter cancerogenus* ATCC 35316
YP_003943705.1	*Enterobacter cloacae* SCF1
YP_001178651.1	*Enterobacter sp.* 638
YP_003529585.1	*Erwinia amylovora* CFBP1430
YP_003739639.1	*Erwinia billingiae* Eb661
YP_002647274.1	*Erwinia pyrifoliae* Ep1/96
YP_001906204.1	*Erwinia tasmaniensis* Et1/99
AAA67647.1	*Escherichia coli* str. K-12 substr. MG1655
YP_003911304.1	*Ferrimonas balearica* DSM 9799
ZP_06050963.1	*Grimontia hollisae* CIP 101886
YP_002241087.1	*Klebsiella pneumoniae* 342
YP_001340351.1	*Marinomonas sp.* MED121
YP_003518500.1	*Pantoea ananatis* LMG 20103
ZP_07380067.1	*Pantoea sp.* aB
ZP_05730718.1	*Pantoea sp.* At-9b

YP_003933027.1	*Pantoea vagans* C9-1
YP_048339.1	*Pectobacterium atrosepticum* SCRI1043
YP_003261561.1	*Pectobacterium wasabiae* WPP163
YP_128324.1	*Photobacterium profundum* SS9
ZP_01161142.1	*Photobacterium sp.* SKA34
YP_003042698.1	*Photorhabdus asymbiotica*
NP_931571.1	*Photorhabdus luminescens laumondii* TTO1
YP_002153230.1	*Proteus mirabilis* HI4320
ZP_03318468.1	*Providencia alcalifaciens* DSM 30120
ZP_06125601.1	*Providencia rettgeri* DSM 1131
ZP_05974162.1	*Providencia rustigianii* DSM 4541
ZP_02958890.1	*Providencia stuartii* ATCC 25827
ZP_01114933.1	*Reinekea sp.* MED297
ZP_06192599.1	*Serratia odorifera* 4Rx13
YP_001476504.1	*Serratia proteamaculans* 568
YP_925918.1	*Shewanella amazonensis* SB2B
YP_001552466.1	*Shewanella baltica* OS195
ZP_02156419.1	*Shewanella benthica* KT99
YP_561039.1	*Shewanella denitrificans* OS217
YP_748718.1	*Shewanella frigidimarina* NCIMB 400
YP_001672255.1	*Shewanella halifaxensis* HAW-EB4
YP_001092155.1	*Shewanella loihica* PV-4
NP_715667.1	*Shewanella oneidensis* MR-1
YP_001499886.1	*Shewanella pealeana* ATCC 700345
YP_002309484.1	*Shewanella piezotolerans* WP3
ZP_01707419.1	*Shewanella putrefaciens* 200
YP_001471768.1	*Shewanella sediminis* HAW-EB3
YP_867679.1	*Shewanella sp.* ANA-3
YP_961424.1	*Shewanella sp.* W3-18-1
YP_003554805.1	*Shewanella violacea* DSS12
YP_001758421.1	*Shewanella woodyi* ATCC 51908
YP_312780.1	*Shigella sonnei* Ss046
YP_453801.1	*Sodalis glossinidius* str. 'morsitans'
YP_003072465.1	*Teredinibacter turnerae* T7901
ZP_06179380.1	*Vibrio alginolyticus* 40B
ZP_01236905.1	*Vibrio angustum* S14
ZP_07742018.1	*Vibrio caribbenthicus* ATCC BAA-2122
ZP_04409571.1	*Vibrio cholerae* TM 11079-80
ZP_05883850.1	*Vibrio coralliilyticus* ATCC BAA-450
ZP_05879944.1	*Vibrio furnissii* CIP 102972
ZP_06177053.1	*Vibrio harveyi* 1DA3
ZP_05883428.1	*Vibrio metschnikovii* CIP 69.14
ZP_05943245.1	*Vibrio orientalis* CIP 102891
ZP_05121178.1	*Vibrio parahaemolyticus* 16
ZP_01870130.1	*Vibrio shilonii* AK1
ZP_05927574.1	*Vibrio sp.* RC341
ZP_00992840.1	*Vibrio splendidus* 12B01
NP_759941.1	*Vibrio vulnificus* CMCP6
ZP_01815878.1	*Vibrionales bacterium* SWAT-3
NP_871126.1	*Wigglesworthia glossinidia* endosymbiont of *Glossina brevipalpis*
YP_003466467.1	*Xenorhabdus bovienii* SS-2004
YP_003713987.1	*Xenorhabdus nematophila* ATCC 19061
ZP_04620751.1	*Yersinia aldovae* ATCC 35236

ZP_04628380.1	*Yersinia bercovieri* ATCC 43970
ZP_04634369.1	*Yersinia frederiksenii* ATCC 33641
ZP_04637122.1	*Yersinia intermedia* ATCC 29909
ZP_04624653.1	*Yersinia kristensenii* ATCC 33638
ZP_04641541.1	*Yersinia mollaretii* ATCC 43969
NP_667807.1	*Yersinia pestis* Pestoides F
ZP_04612258.1	*Yersinia rohdei* ATCC 43380
ZP_04616537.1	*Yersinia ruckeri* ATCC 29473

GenBank accession numbers (except for *C. fasciculata*, which has the TriTrypDB scaffold number and coordinates) in bold typeface were sequenced in this work. The proteins from *C.* K. crithidii and *C.* K. desouzaii were not used in phylogenetic analyses.
