# Supplementary material for: Identification and Phylogenetic Analysis of Heme Synthesis Genes in Trypanosomatids and Their Bacterial Endosymbionts
Source: PLoS One. 2011 Aug 10;6(8):e23518. doi: 10.1371/journal.pone.0023518 (PMC3154472; doi:10.1371/journal.pone.0023518)
Supplement: Table S11 — Proteins utilized in the phylogenetic analysis of ferrochelatase (FeCH) and the respective organism names. (PDF) [file pone.0023518.s024.pdf]

**Accession number**	**Organism**
**JF756597**	*Candidatus* Kinetoplastibacterium blastocrithidii
**JF756598**	*Candidatus* Kinetoplastibacterium crithidii
**JF756599**	*Candidatus* Kinetoplastibacterium galatii
**JF756600**	*Candidatus* Kinetoplastibacterium oncopeltii
**JF756651**	*Angomonas deanei* TCC036E
**JF756650**	*Crithidia acanthocephali* TCC037E
scf7180000113895:5000..1164	*Crithidia fasciculata* Cf-C1
**JF756653**	*Endotrypanum schaudinni* TCC224
**JF756655**	*Herpetomonas muscarum* TCC001E
XP_001563927.1	*Leishmania braziliensis* MHOM/BR/75/M2904
XP_001464815.1	*Leishmania infantum* JPCM5
XP_001682405	*Leishmania major* Friedlin
**JF756654**	*Leptomonas costaricensis* TCC169E
**JF756658**	*Parabodo caudatus* ATCC30905
**JF756656**	*Phytomonas sp.* Jma TCC066
**JF756649**	*Strigomonas culicis* TCC012E
**JF756657**	*Strigomonas galati* TCC219
**JF756652**	*Strigomonas oncopelti* TCC290E
YP_003189026.1	*Acetobacter pasteurianus* IFO 3283-01
ZP_06686166.1	*Achromobacter piechaudii* ATCC 43553
YP_003977224	*Achromobacter xylosoxidans* A8
YP_002218819.1	*Acidithiobacillus ferrooxidans* ATCC 53993
YP_002755471.1	*Acidobacterium capsulatum* ATCC 51196
YP_873165.1	*Acidothermus cellulolyticus* 11B
ZP_04764699.1	*Acidovorax delafieldii* 2AN
ZP_04660195.1	*Acinetobacter baumannii* AB900
ZP_05629556.1	*Actinobacillus minor* 202
ZP_03924685.1	*Actinomyces coleocanis* DSM 15436
ZP_07361040.1	*Actinomyces viscosus* C505
YP_001141652.1	*Aeromonas salmonicida salmonicida* A449
ZP_07025363.1	*Afipia sp.* 1NLS2
YP_003256160.1	*Aggregatibacter actinomycetemcomitans* D11S-1
YP_002545610.1	*Agrobacterium radiobacter* K84
YP_002550680	*Agrobacterium vitis* S4
ZP_07375331.1	*Ahrensia sp.* R2A130
YP_692014.1	*Alcanivorax borkumensis* SK2
ZP_07722431.1	*Algoriphagus sp.* PR1
ZP_07020298.1	*Alicycliphilus denitrificans* BC
ZP_03493091.1	*Alicyclobacillus acidocaldarius* LAA1
YP_002262324.1	*Aliivibrio salmonicida* LFI1238
YP_741581.1	*Alkalilimnicola ehrlichii* MLHE-1
YP_003442400.1	*Allochromatium vinosum* DSM 180
YP_002124966.1	*Alteromonas macleodii* 'Deep ecotype'
YP_002316621.1	*Anoxybacillus flavithermus* WK1
NP_213646.1	*Aquifex aeolicus* VF5
NP_001031941.1	*Arabidopsis thaliana*
YP_003697464.1	*Arcanobacterium haemolyticum* DSM 20595
YP_159744.1	*Aromatoleum aromaticum* EbN1
CBA71691.1	*Arsenophonus nasoniae*
YP_003917338.1	*Arthrobacter arilaitensis* Re117
XP_001396807.1	*Aspergillus niger* CBS 513.88

ZP_01226804.1	*Aurantimonas manganoxydans* SI85-9A1
YP_934086.1	*Azoarcus sp.* BH72
YP_001527188.1	*Azorhizobium caulinodans* ORS 571
YP_002801245.1	*Azotobacter vinelandii* DJ
ZP_06365251.1	*Bacillus cellulosilyticus* DSM 2522
YP_004207023.1	*Bacillus subtilis* BSn5
CBW26948.1	*Bacteriovorax marinus* SJ
NP_970190.1	*Bdellovibrio bacteriovorus* HD100
ZP_02002886.1	*Beggiatoa sp.* PS
YP_001834522.1	*Beijerinckia indica indica* ATCC 9039
ZP_01088523.1	*Blastopirellula marina* DSM 3645
YP_787218.1	*Bordetella avium* 197N
NP_890472.1	*Bordetella bronchiseptica* RB50
NP_885649.1	*Bordetella parapertussis* 12822
NP_881130.1	*Bordetella pertussis* Tohama I
YP_001630161.1	*Bordetella petrii* DSM 12804
NP_776479.1	*Bos taurus*
NP_774392.1	*Bradyrhizobium japonicum* USDA 110
YP_002771296.1	*Brevibacillus brevis* NBRC 100599
AAK14798.1	*Brucella abortus*
YP_001807376.1	*Burkholderia ambifaria* MC40-6
YP_003165780.1	*Candidatus* Accumulibacter phosphatis clade IIA str. UW-1
YP_002923123.1	*Candidatus* Hamiltonella defensa 5AT (Acyrthosiphon pisum)
YP_003064613.1	*Candidatus* Liberibacter asiaticus str. psy62
EFD93161.1	*Candidatus* Parvarchaeum acidophilus ARMAN-5
YP_008556.1	*Candidatus* Protochlamydia amoebophila UWE25
YP_003550405.1	*Candidatus* Puniceispirillum marinum IMCC1322
ZP_07396246.1	*Candidatus* Regiella insecticola LSR1
YP_903747.1	*Candidatus* Ruthia magnifica str. Cm (*Calyptogena magnifica*)
YP_001219321.1	*Candidatus* Vesicomyosocius okutanii HA
ZP_04057978.1	*Capnocytophaga gingivalis* ATCC 33624
ZP_05704189.1	*Cardiobacterium hominis* ATCC 15826
YP_003112386.1	*Catenulispora acidiphila* DSM 44928
ZP_04450425.1	*Catonella morbi* ATCC 51271
YP_001984113.1	*Cellvibrio japonicus* Ueda107
YP_003126103.1	*Chitinophaga pinensis* DSM 2588
EFN54521.1	*Chlorella variabilis*
NP_902150.1	*Chromobacterium violaceum* ATCC 12472
YP_572953.1	*Chromohalobacter salexigens* DSM 3043
ZP_07086081.1	*Chryseobacterium gleum* ATCC 35910
ZP_05782783.1	*Citreicella sp.* SE45
YP_003364167.1	*Citrobacter rodentium* ICC168
ZP_06861135.1	*Citromicrobium bathyomarinum* JL354
YP_267788.1	*Colwellia psychrerythraea* 34H
YP_003277282.1	*Comamonas testosteroni* CNB-2
ZP_01102046.1	*Congregibacter litoralis* KT71
YP_003549603.1	*Coraliomargarita akajimensis* DSM 45221
NP_939632.1	*Corynebacterium diphtheriae* NCTC 13129
NP_819097.2	*Coxiella burnetii* RSA 493
YP_003715496.1	*Croceibacter atlanticus* HTCC2559
YP_001438854.1	*Cronobacter sakazakii* ATCC BAA-894
YP_003151035.1	*Cryptobacterium curtum* DSM 15641
XP_003194818.1	*Cryptococcus gattii* WM276

YP_002005146.1	*Cupriavidus taiwanensis*
CBA32822.1	*Curvibacter* putative symbiont of *Hydra magnipapillata*
ZP_05044143.1	*Cyanobium sp.* PCC 7001
YP_001803982.1	*Cyanothece sp.* ATCC 51142
YP_680066.1	*Cytophaga hutchinsonii* ATCC 33406
NP_571706.1	*Danio rerio*
YP_284155.1	*Dechloromonas aromatica* RCB
NP_294855.1	*Deinococcus radiodurans* R1
YP_001563585.1	*Delftia acidovorans* SPH-1
YP_003691237.1	*Desulfurivibrio alkaliphilus* AHT2
YP_001210125.1	*Dichelobacter nodosus* VCS1703A
YP_003332655.1	*Dickeya dadantii* Ech586
YP_001534832.1	*Dinoroseobacter shibae* DFL 12
ZP_01049599.1	*Dokdonia donghaensis* MED134
XP_002070617.1	*Drosophila willistoni*
YP_003088784.1	*Dyadobacter fermentans* DSM 18053
ZP_06713857.1	*Edwardsiella tarda* ATCC 23685
YP_003181918.1	*Eggerthella lenta* DSM 2243
ZP_03712668.1	*Eikenella corrodens* ATCC 23834
ZP_05621008.1	*Enhydrobacter aerosaccus* SK60
YP_003942844.1	*Enterobacter cloacae* SCF1
ZP_05563472.1	*Enterococcus faecalis* DS5
YP_003739199.1	*Erwinia billingiae* Eb661
YP_459152.1	*Erythrobacter litoralis* HTCC2594
ZP_02902346.1	*Escherichia albertii* TW07627
YP_002886830.1	*Exiguobacterium sp.* AT1b
YP_003912471.1	*Ferrimonas balearica* DSM 9799
ZP_05571281.1	*Ferroplasma acidarmanus* fer1
YP_001193292.1	*Flavobacterium johnsoniae* UW101
ZP_04990009.1	*Francisella novicida* GA99-3548
YP_714703.1	*Frankia alni* ACN14a
ZP_01439734.1	*Fulvimarina pelagi* HTCC2506
YP_003847930.1	*Gallionella capsiferriformans* ES-2
AAB66503.1	*Gallus gallus*
ZP_04776463.1	*Gemella haemolysans* ATCC 10379
ZP_02735861.1	*Gemmata obscuriglobus* UQM 2246
YP_002762295.1	*Gemmatimonas aurantiaca* T-27
YP_146515.1	*Geobacillus kaustophilus* HTA426
YP_001601578.1	*Gluconacetobacter diazotrophicus* PAI 5
YP_191303.1	*Gluconobacter oxydans* 621H
YP_863230.1	*Gramella forsetii* KT0803
ZP_05851527.1	*Granulicatella elegans* ATCC 700633
ZP_06054046.1	*Grimontia hollisae* CIP 101886
CBW29505.1	*Haemophilus influenzae* 10810
YP_431972.1	*Hahella chejuensis* KCTC 2396
YP_003270053.1	*Haliangium ochraceum* DSM 14365
YP_003898589.1	*Halomonas elongata* DSM 2581
YP_001002397.1	*Halorhodospira halophila* SL1
YP_003261958.1	*Halothiobacillus neapolitanus* c2
ZP_04580831.1	*Helicobacter bilis* ATCC 43879
YP_003774040.1	*Herbaspirillum seropedicae* SmR1
YP_001100893.1	*Herminiimonas arsenicoxydans*
YP_001546311.1	*Herpetosiphon aurantiacus* ATCC 23779

ZP_02165952.1	*Hoeflea phototrophica* DFL-43
BAA00628.1	*Homo sapiens*
ZP_02177991.1	*Hydrogenivirga sp.* 128-5-R1-1
YP_003432005.1	*Hydrogenobacter thermophilus* TK-6
YP_002122164.1	*Hydrogenobaculum sp.* Y04AAS1
YP_003756777.1	*Hyphomicrobium denitrificans* ATCC 51888
ZP_01042955.1	*Idiomarina baltica* OS145
YP_004178356.1	*Isosphaera pallida* ATCC 43644
YP_508235.1	*Jannaschia sp.* CCS1
YP_001354575.1	*Janthinobacterium sp.* Marseille
YP_003146227.1	*Kangiella koreensis* DSM 16069
YP_003965102.1	*Ketogulonicigenium vulgare* Y25
YP_001361302.1	*Kineococcus radiotolerans* SRS30216
ZP_04601673.1	*Kingella oralis* ATCC 51147
BAJ31205.1	*Kitasatospora setae* KM-6054
YP_002240023.1	*Klebsiella pneumoniae* 342
ZP_02161937.1	*Kordia algicida* OT-1
YP_003381625.1	*Kribbella flavida* DSM 17836
ZP_06972781.1	*Ktedonobacter racemifer* DSM 44963
ZP_05116035.1	*Labrenzia alexandrii* DFL-11
ZP_05745049.1	*Lactobacillus antri* DSM 16041
ZP_05743641.1	*Lactobacillus iners* DSM 13335
YP_003354110.1	*Lactococcus lactis lactis* KF147
YP_002796395.1	*Laribacter hongkongensis* HLHK9
YP_003998063.1	*Leadbetterella byssophila* DSM 17132
ZP_01059609.1	*Leeuwenhoekiella blandensis* MED217
YP_122830.1	*Legionella pneumophila* str. Paris
ZP_01877243.1	*Lentisphaera araneosa* HTCC2155
EES52000.1	*Leptospirillum ferrodiazotrophum*
YP_001791595.1	*Leptothrix cholodnii* SP-6
ZP_03914598.1	*Leuconostoc mesenteroides cremoris* ATCC 19254
ZP_01914472.1	*Limnobacter sp.* MED105
NP_471646.1	*Listeria innocua* Clip11262
ZP_01002254.1	*Loktanella vestfoldensis* SKA53
ZP_03697214.1	*Lutiella nitroferrum* 2002
ZP_07050854.1	*Lysinibacillus fusiformis* ZC1
YP_002560952.1	*Macrococcus caseolyticus* JCSC5402
YP_865616.1	*Magnetococcus sp.* MC-1
ZP_04978366.1	*Mannheimia haemolytica* PHL213
YP_958768.1	*Marinobacter aquaeolei* VT8
YP_001341072.1	*Marinomonas sp.* MWYL1
ZP_01012530.1	*Maritimibacter alkaliphilus* HTCC2654
YP_004054683.1	*Marivirga tractuosa* DSM 4126
YP_003507187.1	*Meiothermus ruber* DSM 1279
ABF50742.1	*Mesocricetus auratus*
NP_104219.1	*Mesorhizobium loti* MAFF303099
YP_001021685.1	*Methylibium petroleiphilum* PM1
YP_544881.1	*Methylobacillus flagellatus* KT
ZP_07652810.1	*Methylobacter tundripaludum* SV96
YP_001638942.1	*Methylobacterium extorquens* PA1
YP_002363938.1	*Methylocella silvestris* BL2
YP_114114.1	*Methylococcus capsulatus* str. Bath
ZP_05104653.1	*Methylophaga thiooxidans* DMS010

ZP_06886810.1	*Methylosinus trichosporium* OB3b
YP_003049146.1	*Methylotenera mobilis* JLW8
YP_003051656.1	*Methylovorus sp.* SIP3-4
YP_001655979.1	*Microcystis aeruginosa* NIES-843
YP_003837328.1	*Micromonospora aurantiaca* ATCC 27029
ZP_01690083.1	*Microscilla marina* ATCC 23134
YP_003626490.1	*Moraxella catarrhalis* RH4
ZP_01896793.1	*Moritella sp.* PE36
ZP_07748083.1	*Mucilaginibacter paludis* DSM 18603
AAA37615.1	*Mus musculus*
YP_001134918.1	*Mycobacterium gilvum* PYR-GCK
YP_629789.1	*Myxococcus xanthus* DK 1622
NP_273760.1	*Neisseria meningitidis* MC58
ZP_05978462.1	*Neisseria mucosa* ATCC 25996
ZP_05984461.1	*Neisseria subflava* NJ9703
XP_001632930.1	*Nematostella vectensis*
YP_578605.1	*Nitrobacter hamburgensis* X14
ZP_01126444.1	*Nitrococcus mobilis* Nb-231
YP_003526615.1	*Nitrosococcus halophilus* Nc4
NP_841517.1	*Nitrosomonas europaea* ATCC 19718
YP_747005.1	*Nitrosomonas eutropha* C91
YP_413105.1	*Nitrosospira multiformis* ATCC 25196
YP_924102.1	*Nocardioides sp.* JS614
YP_001866010.1	*Nostoc punctiforme* PCC 73102
YP_498605.1	*Novosphingobium aromaticivorans* DSM 12444
ZP_02152442.1	*Oceanibulbus indolifex* HEL-45
ZP_00998988.1	*Oceanicola batsensis* HTCC2597
YP_004057680.1	*Oceanithermus profundus* DSM 14977
NP_692089.1	*Oceanobacillus iheyensis* HTE831
ZP_01306620.1	*Oceanobacter sp.* RED65
ZP_01167499.1	*Oceanospirillum sp.* MED92
ZP_04682825.1	*Ochrobactrum intermedium* LMG 3301
ZP_05050716.1	*Octadecabacter antarcticus* 307
YP_002287926.1	*Oligotropha carboxidovorans* OM5
YP_001819352.1	*Opitutus terrae* PB90-1
ZP_02328676.1	*Paenibacillus larvae larvae* BRL-230010
ZP_07377424.1	*Pantoea sp.* aB
YP_916366.1	*Paracoccus denitrificans* PD1222
YP_003854109.1	*Parvularcula bermudensis* HTCC2503
ZP_05920819.1	*Pasteurella dagmatis* ATCC 43325
YP_002049510.1	*Paulinella chromatophora*
YP_049287.1	*Pectobacterium atrosepticum* SCRI1043
YP_003093870.1	*Pedobacter heparinus* DSM 2366
ZP_01441700.1	*Pelagibaca bermudensis* HTCC2601
YP_002731437.1	*Persephonella marina* EX-H1
ZP_02145373.1	*Phaeobacter gallaeciensis* BS107
XP_002179541.1	*Phaeodactylum tricornutum* CCAP 1055/1
XP_001803227.1	*Phaeosphaeria nodorum* SN15
YP_129238.1	*Photobacterium profundum* SS9
YP_003042212.1	*Photorhabdus asymbiotica*
YP_023726.1	*Picrophilus torridus* DSM 9790
YP_003370976.1	*Pirellula staleyi* DSM 6068
ZP_01857739.1	*Planctomyces maris* DSM 8797

ZP_08094640.1	*Planococcus donghaensis* MPA1U2
CAC82988.1	*Plasmodium falciparum*
CAD12105.1	*Plasmodium yoelii*
ZP_01905842.1	*Plesiocystis pacifica* SIR-1
ZP_01117850.1	*Polaribacter irgensii* 23-P
YP_001798192.1	*Polynucleobacter necessarius necessarius* STIR1
EFA74915.1	*Polysphondylium pallidum* PN500
NP_001126366.1	*Pongo abelii*
NP_904470.1	*Porphyromonas gingivalis* W83
YP_001014409.1	*Prochlorococcus marinus* str. NATL1A
YP_003688804.1	*Propionibacterium freudenreichii shermanii* CIRM-BIA1
YP_002151902.1	*Proteus mirabilis* HI4320
ZP_06125476.1	*Providencia rettgeri* DSM 1131
YP_663541.1	*Pseudoalteromonas atlantica* T6c
YP_001350636.1	*Pseudomonas aeruginosa* PA7
YP_606608.1	*Pseudomonas entomophila* L48
YP_002870420.1	*Pseudomonas fluorescens* SBW25
YP_001186576.1	*Pseudomonas mendocina* ymp
ADR58476.1	*Pseudomonas putida* BIRD-1
YP_001172484.1	*Pseudomonas stutzeri* A1501
NP_790962.1	*Pseudomonas syringae* pv. tomato str. DC3000
ZP_05086468.1	*Pseudovibrio sp.* JE062
YP_263679.1	*Psychrobacter arcticus* 273-4
ZP_01252533.1	*Psychroflexus torquis* ATCC 700755
YP_943855.1	*Psychromonas ingrahamii* 37
EFQ91097.1	*Pyrenophora teres* f. teres 0-1
YP_725643.1	*Ralstonia eutropha* H16
EDM14670.1	*Rattus norvegicus*
ZP_01113141.1	*Reinekea sp.* MED297
YP_001624374.1	*Renibacterium salmoninarum* ATCC 33209
YP_001979926.1	*Rhizobium etli* CIAT 652
YP_354280.1	*Rhodobacter sphaeroides* 2.4.1
YP_002526955.1	Rhodobacter sphaeroides KD131
YP_522808.1	*Rhodoferax ferrireducens* T118
YP_004012040.1	*Rhodomicrobium vannielii* ATCC 17100
YP_001989976.1	*Rhodopseudomonas palustris* TIE-1
YP_002299712.1	*Rhodospirillum centenum* SW
ZP_02061957.1	*Rickettsiella grylli*
YP_004046068.1	*Riemerella anatipestifer* DSM 15868
YP_003194216.1	*Robiginitalea biformata* HTCC2501
ZP_07659792.1	*Roseibium sp.* TrichSKD4
YP_001432135.1	*Roseiflexus castenholzii* DSM 13941
ZP_02140439.1	*Roseobacter litoralis* Och 149
ZP_06897188.1	*Roseomonas cervicalis* ATCC 49957
ZP_00959476.1	*Roseovarius nubinhibens* ISM
YP_165347.1	*Ruegeria pomeroyi* DSS-3
YP_525605.1	*Saccharophagus degradans* 2-40
ZP_01745881.1	*Sagittula stellata* E-37
YP_001159838.1	*Salinispora tropica* CNB-440
YP_003313619.1	*Sanguibacter keddieii* DSM 10542
ZP_06756399.1	*Scardovia inopinata* F0304
XP_002574902.1	*Schistosoma mansoni*
YP_003659790.1	*Segniliparus rotundus* DSM 44985

ZP_06191885.1	*Serratia odorifera* 4Rx13
YP_927193.1	*Shewanella amazonensis* SB2B
YP_003523671.1	*Sideroxydans lithotrophicus* ES-1
ZP_05785696.1	*Silicibacter lacuscaerulensis* ITI-1157
ZP_06753699.1	*Simonsiella muelleri* ATCC 29453
YP_001328356.1	*Sinorhizobium medicae* WSM419
ZP_06160431.1	*Slackia exigua* ATCC 700122
YP_454374.1	*Sodalis glossinidius* str. 'morsitans'
YP_001612320.1	*Sorangium cellulosum* 'So ce 56'
YP_003320569.1	*Sphaerobacter thermophilus* DSM 20745
ZP_03968961.1	*Sphingobacterium spiritivorum* ATCC 33300
ZP_07575244.1	*Sphingobium chlorophenolicum* L-1
YP_001265074.1	*Sphingomonas wittichii* RW1
YP_617295.1	*Sphingopyxis alaskensis* RB2256
YP_003385511.1	*Spirosoma linguale* DSM 74
YP_301056.1	*Staphylococcus saprophyticus saprophyticus* ATCC 15305
ZP_01545825.1	*Stappia aggregata* IAM 12614
YP_003695760.1	*Starkeya novella* DSM 506
YP_001974272.1	*Stenotrophomonas maltophilia* K279a
ZP_01459698.1	*Stigmatella aurantiaca* DW4/3-1
NP_722354.1	*Streptococcus mutans* UA159
YP_001035270.1	*Streptococcus sanguinis* SK36
ZP_06908974.1	*Streptomyces pristinaespiralis* ATCC 25486
YP_003342229.1	*Streptosporangium roseum* DSM 43021
ZP_00954522.1	*Sulfitobacter sp.* EE-36
YP_393431.1	*Sulfurimonas denitrificans* DSM 1251
YP_001358976.1	*Sulfurovum sp.* NBC37-1
NP_001163994.1	*Sus scrofa*
YP_073989.1	*Symbiobacterium thermophilum* IAM 14863
YP_731203.1	*Synechococcus sp.* CC9311
YP_003075593.1	*Teredinibacter turnerae* T7901
ZP_05342562.1	*Thalassiobium sp.* R2A62
EED96635.1	*Thalassiosira pseudonana* CCMP1335
YP_002355372.1	*Thauera sp.* MZ1T
YP_004101607.1	*Thermaerobacter marianensis* DSM 12885
YP_003324302.1	*Thermobaculum terrenum* ATCC BAA-798
YP_290001.1	*Thermobifida fusca* YX
YP_002522588.1	*Thermomicrobium roseum* DSM 5159
YP_003299304.1	*Thermomonospora curvata* DSM 43183
NP_393791.1	*Thermoplasma acidophilum* DSM 1728
ZP_03495934.1	*Thermus aquaticus* Y51MC23
YP_003459504.1	*Thioalkalivibrio sp.* K90mix
YP_315791.1	*Thiobacillus denitrificans* ATCC 25259
YP_392128.1	*Thiomicrospira crunogena* XCL-2
YP_003643370.1	*Thiomonas intermedia* K12
YP_002893397.1	*Tolumonas auensis* DSM 9187
XP_002365079.1	*Toxoplasma gondii* ME49
XP_970181.1	*Tribolium castaneum*
YP_002944317.1	*Variovorax paradoxus* S110
ZP_04600320.1	*Veillonella dispar* ATCC 17748
YP_996351.1	*Verminephrobacter eiseniae* EF01-2
ZP_05059070.1	*Verrucomicrobiae bacterium* DG1235
ZP_01235835.1	*Vibrio angustum* S14

YP_003709810.1	*Waddlia chondrophila* WSU 86-1044
NP_871532.1	*Wigglesworthia glossinidia* endosymbiont of *Glossina brevipalpis*
YP_001417711.1	*Xanthobacter autotrophicus* Py2
YP_003374601.1	*Xanthomonas albilineans*
XP_002934473.1	*Xenopus (Silurana) tropicalis*
YP_003467510.1	*Xenorhabdus bovienii* SS-2004
YP_003327196.1	*Xylanimonas cellulosilytica* DSM 15894
NP_297856.1	*Xylella fastidiosa* 9a5c
YP_001164088.1	*Yersinia pestis* Pestoides F
YP_003582675.1	*Zunongwangia profunda* SM-A87
ZP_04758959.1	*Zymomonas mobilis mobilis* ATCC 10988

GenBank accession numbers (except for *C. fasciculata*, which has the TriTrypDB scaffold number and coordinates) in bold typeface were sequenced in this work.
